# Gut microbiota‐derived tryptophan metabolites improve total parenteral nutrition‐associated infections by regulating Group 3 innate lymphoid cells

**DOI:** 10.1002/imt2.70007

**Published:** 2025-02-26

**Authors:** Longchang Huang, Peng Wang, Shuai Liu, Guifang Deng, Xin Qi, Guangming Sun, Xuejin Gao, Li Zhang, Yupeng Zhang, Yaqin Xiao, Tingting Gao, Gulisudumu Maitiabula, Xinying Wang

**Affiliations:** ^1^ Department of General Surgery Jinling Hospital, Medical School of Nanjing University Nanjing China; ^2^ Department of Digestive Disease Research Center Gastrointestinal Surgery, The First People's Hospital of Foshan Foshan China; ^3^ Department of Clinical Nutrition Union Shenzhen Hospital of Huazhong University of Science and Technology Shenzhen China

**Keywords:** Group 3 innate lymphoid cells, gut microbiota, indole‐3‐carboxylic acid, intestinal barrier integrity, lactobacillus murinus, TPN‐associated infection

## Abstract

Clinical nutritional support is recognized by Klinefner's Surgery as one of the four pivotal advancements in surgical practice during the 20th century. Surgeons regard clinical nutrition as a “life‐saving” discipline, pivotal in preserving the lives of numerous critically ill patients and facilitating the success of many surgical procedures. Parenteral nutrition (PN) support serves as a crucial component of clinical nutritional therapy, while a range of complications associated with total parenteral nutrition (TPN) can significantly undermine the efficacy of patient treatment. Impaired intestinal homeostasis is strongly associated with the occurrence and progression of TPN‐related infections, yet the underlying mechanisms remain poorly understood. In this study, RNA sequencing and single‐cell RNA sequencing (scRNA‐Seq) revealed that reduced secretion of interleukin‐22 (IL‐22) by intestinal Group 3 innate lymphoid cells (ILC3s) is a significant factor contributing to the onset of TPN‐related infections. Additionally, through 16S ribosomal RNA (16S rRNA) gene sequencing of the gut microbiota from patients with chronic intestinal failure and metagenomic sequencing analysis of the gut microbiota from mice, we observed that TPN reduced the abundance of *Lactobacillus murinus* (*L. murinus*), while supplementation with *L. murinus* could promote IL‐22 secretion by ILC3s. Mechanistically, *L. murinus* upregulates indole‐3‐carboxylic acid, which activates the nuclear receptor Rorγt to stimulate IL‐22 secretion by ILC3s. This pathway strengthens gut barrier integrity and reduces infection susceptibility. Our findings enhance our understanding of the mechanisms driving the onset of TPN‐related infections, highlighting the critical role of gut microbiota in maintaining immune homeostasis and improving clinical outcomes.

## Highlights

- Dysbiosis caused by total parenteral nutrition (TPN) reduces the response of intestinal Group 3 innate lymphoid cells (ILC3s), thereby increasing susceptibility to infection.
- *L. murinus* is reduced in patients with chronic intestinal failure using TPN, and *L. murinus* and its metabolite, indole‐3‐carboxylic acid (ICA), can modulate ILC3s and mitigate infections.
- ICA enhances the secretion of IL‐22 in ILC3s through activation of Rorγt.

## Graphical abstract

During the use of total parenteral nutrition (TPN), the decrease in the immune function of intestinal Group 3 innate lymphoid cells (ILC3s) can lead to an increased susceptibility to infections in patients. Specifically, the use of TPN causes dysbiosis of the gut microbiota, inhibiting the secretion of IL‐22 by intestinal ILC3s, which in turn results in damage to the intestinal barrier. The reduction of *Lactobacillus murinus* (*L. murinus*) is an important factor in this process. Supplementing with *L. murinus* can increase the expression of its metabolite, indole‐3‐carboxylic acid (ICA). ICA promotes the secretion of IL‐22 by ILC3s by targeting Rorγt, thereby improving the intestinal barrier and reducing susceptibility to infections. This study provides an important theoretical basis for using gut microbiota to regulate immune homeostasis in the treatment of clinical diseases.

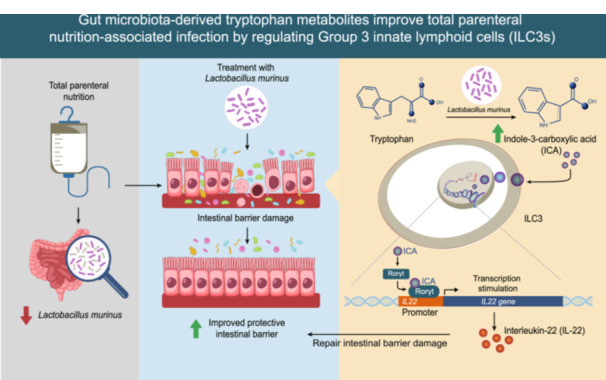

## INTRODUCTION

Parenteral nutrition (PN) is a vital intervention for sustaining patients with chronic intestinal failure (CIF) [1]. When PN provides over 80% of the body's energy requirements, it is termed total parenteral nutrition (TPN) [2]. However, TPN can compromise the integrity of the intestinal mucosal barrier [3, 4], facilitating intestinal bacteria and endotoxin translocation [5], which results in severe, life‐threatening complications [6, 7, 8]. The incidence of TPN‐associated infections can reach up to 42.05% [7]. Previous studies have indicated that patients with CIF receiving TPN have a survival rate of just 58% at 1.5 years, with infection being a major contributor to mortality [9, 10]. Since the precise mechanism underlying TPN‐related infections remains unclear and effective therapies are lacking, investigating their pathogenesis is crucial to improving the prognosis of patients with CIF receiving TPN.

The gut microbiota and their metabolites are associated with various diseases and are potential therapeutic targets [11, 12, 13]. The gut microbiota regulates the host's immune homeostasis by producing indoles, bile acids, and short‐chain fatty acids (SCFAs) [12]. These metabolites bind to and activate pathways, including those involving aryl hydrocarbon receptor (AHR), G protein‐coupled receptor, and Farnesoid X receptor, stimulating the recruitment of macrophages, T cells, and associated cytokines to repair intestinal barrier function, reducing inflammation [14, 15, 16]. A study reported a decrease in Firmicutes abundance and an increase in Bacteroidetes in the gut microbiota of TPN model mice [17], linking TPN‐related infection to gut microbiota alterations.

Group 3 innate lymphoid cells (ILC3s) effectively repair the intestinal barrier function, integrating cues mediated by the cytokines interleukin (IL)‐23 and IL‐1β. It is derived from enteric glial cells to convey information to intestinal epithelial cells (IECs) and intestinal resident cells, promoting intestinal immune and metabolic homeostasis [18]. ILC3s can be activated by microbial metabolites, such as SCFAs and indole, to produce IL‐22, alleviating colitis [19, 20]. The diminished expression of IL‐22 has also been documented in TPN utilization [17]; however, whether alterations in ILC3s are associated with TPN‐related infections remains unclear. In this study, we investigated the role of ILC3s in TPN‐related infections. Additionally, we clarified the specific mechanism through which indole‐3‐carboxylic acid (ICA) derived by *L. murinus* activates ILC3s.

## RESULTS

### Dysbiosis caused by TPN induces intestinal barrier damage and infection

**Figure 1:**
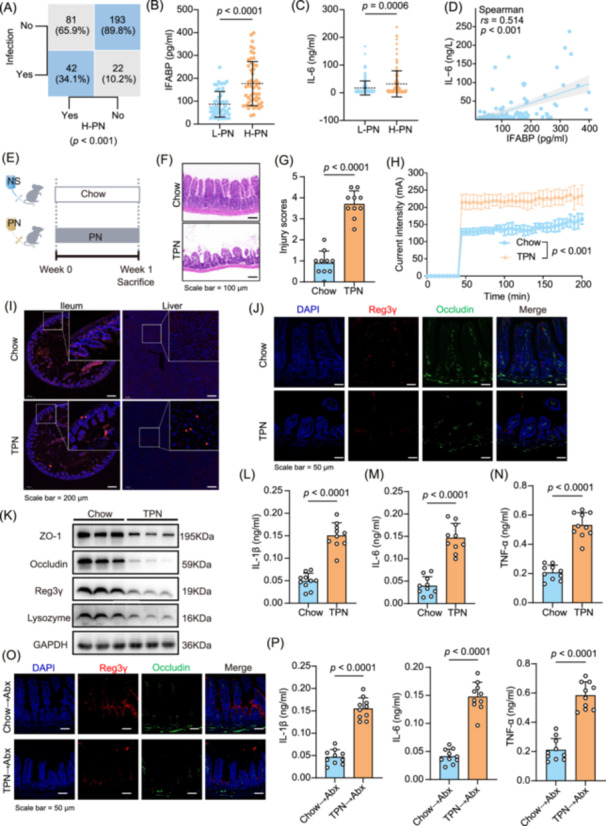
Total parenteral nutrition (TPN) induces infection in humans and mice. (A) Incidence of infections among patients with chronic intestinal failure (CIF) was higher in the H‐PN group. H‐PN, the high‐dose parenteral nutrition group (*n* = 123, PN‐provided energy is > 80% of the required amount). l‐PN, the low‐dose parenteral nutrition group (*n* = 215, PN‐provided energy ≤ 80% of the required amount). (B, C) Serum levels of intestinal fatty acid binding protein (IFABP) (*n* = 52 per group) and Interleukin‐6 (IL‐6) (H‐PN, *n* = 123 and l‐PN, *n* = 215) in patients. (D) Correlation between IFABP and IL‐6 levels in patients. (E) Experimental design for studies on C57BL/6 mice. Representative intestinal hematoxylin and eosin (HE) staining (F) and corresponding injury scores (G) (*n* = 10 mice per group). (H) Measurement of the transmembrane currents of the intestine in mice using an Ussing Chamber (*n* = 5 per group). (I) Immunofluorescence detection of bacteria in the intestines and livers of mice. (J) Immunofluorescence analysis of Reg3γ and Occludin expression in the intestines of mice fed with standard Chow and TPN. (K) Western blot analysis of ZO‐1, Occludin, Reg3γ and Lysozyme expression in the Chow versus TPN groups. GAPDH was used as a loading control. (L) Serum levels of IL‐1β (L), IL‐6 (M), and tumor necrosis factor‐alpha (TNF‐α, N) in the Chow versus TPN groups (*n* = 10 per group). (O) Immunofluorescence analysis of Reg3γ and Occludin expression in the intestine of antibiotic‐treated (Abx) mice posttreatment. (P) Serum levels of IL‐1β, IL‐6, and TNF‐α in the Abx mice after indicated treatments (*n* = 10 per group). *p* values were determined by the Mann–Whitney *U* test (B, C) and the Student's *t*‐test (G, H and L, M).

We retrospectively analyzed 338 patients with CIF undergoing individualized nutritional support to confirm that TPN contributes to intestinal barrier damage and infection (Figure S1). Based on the proportion of PN energy supply, patients were stratified into two groups: the high‐dose parenteral nutrition (H‐PN) group (*n* = 123, PN‐provided energy is >80% of the required amount) and the low‐dose parenteral nutrition (l‐PN) group (*n* = 215, PN‐provided energy ≤80% of the required amount). Through analysis of baseline characteristics, including gender (*p* = 0.585), age (*p* = 0.186), and underlying disease (*p* = 0.322), the two groups did not differ statistically (Table S1). As indicated in Figure 1A, there were more infections in the H‐PN group (34.1% vs. 10.2%, *p* < 0.001). Moreover, patients in the H‐PN group exhibited higher levels of intestinal fatty acid binding protein (IFABP), IL‐6, and C‐reactive protein (CRP) levels (Figure 1B,C and Figure S2A). We also observed a significant positive correlation between the IFABP and IL‐6 levels (Figure 1D), as well as CRP (Figure S2B). These findings suggest that using TPN is associated with clinical adverse events, such as intestinal barrier damage and infections.

Subsequently, we demonstrated gut barrier damage and infection in a mouse model utilizing TPN treatment (Figure 1E). In comparison to the control group, TPN significantly altered the tissue histopathological status, resulting in increased intestinal histological scores, shorter villi, and shallow crypts in TPN group mice (Figure 1F,G, Figure S2C,D). We evaluated the intestinal mechanical barrier of the mouse using the Ussing chamber system and observed that under constant voltage conditions, Chow mice had significantly weaker transmembrane currents than TPN mice (*p* < 0.001), indicating a notable increase in intestinal permeability in the TPN group (Figure 1H). We also labeled intestinal bacteria in mice with Cy5‐labeled type D alanine (Cy5ADA), followed by immunofluorescence experiments. The results revealed that in the TPN group, intestinal bacteria breached the intestinal submucosa and showed significant fluorescence in the liver (Figure 1I), indicating a substantial disruption in the intestinal barrier. Concurrently, we observed a significant downregulation of antimicrobial peptides (Reg3γ, *p* = 0.0019), tight junction proteins (Occludin, *p* = 0.0019 and ZO‐1, *p* = 0.0015), and lysozyme (*p* = 0.0026), all of which are markers of gut barrier integrity (Figure 1J,K and Figure S2E). Using enzyme‐linked immunosorbent assay, we quantified cytokine concentrations in murine blood, demonstrating that TPN administration significantly elevated IL‐1β (*p* < 0.0001), IL‐6 (*p* < 0.0001), and tumor necrosis factor‐alpha (TNF‐α, *p* < 0.0001) levels in the serum (Figure 1L–N). Based on these findings, it appears that TPN damages the intestinal barrier, resulting in infection.

We performed fecal microbiota transplantation (FMT) experiments to elucidate whether the effects above are attributable to alterations in the gut microbiota (Figure S2F). Our findings revealed that mice receiving fecal transplants from TPN‐treated mice exhibited intestinal barrier damage (Figure 1O, Figure S2G–J) and markedly elevated inflammatory markers (Figure 1P, *p* < 0.0001). Conversely, mice receiving fecal transplants from the Chow group did not demonstrate these changes. These results suggest that TPN‐induced modifications in the gut microbiota contribute to intestinal damage and infection.

### Dysbiosis reduces intestinal ILC3s response

**Figure 2:**
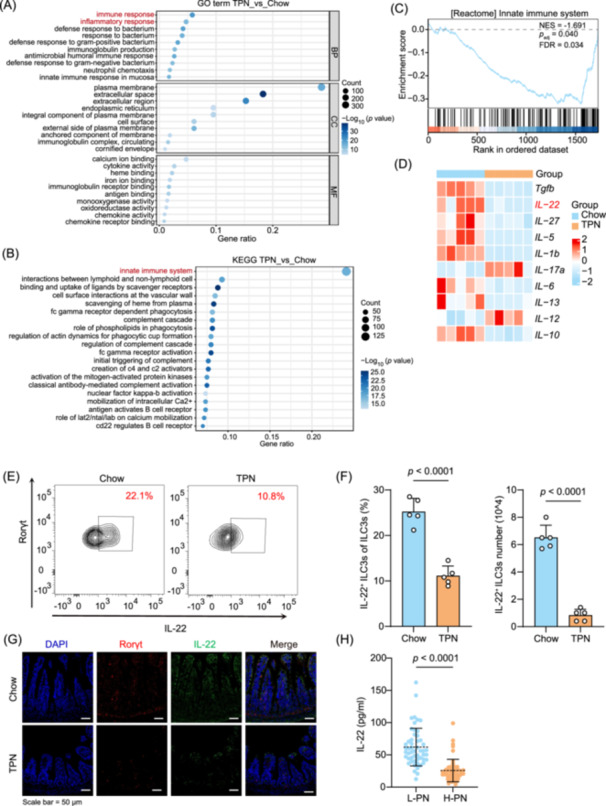
TPN induces a microenvironment characterized by low Group 3 innate lymphoid cells (ILC3s) responses. Gene Ontology (GO) (A) and Kyoto Encyclopedia of Genes and Genomes (KEGG) (B) pathway analyses of mouse intestine tissues (*n* = 5 per group). The two most prominent GO pathways identified are related to immune response and inflammatory response. KEGG pathway analysis revealed significant modifications in the intestinal innate immune system. (C) Gene Set Enrichment Analysis (GSEA) of mouse intestine tissues (*n* = 5 per group). (D) Heatmap depicting differential mRNA expression of cytokines in intestines of mice from the Chow and TPN groups (*n* = 5 per group). Representative fluorescence‐activated cell sorting (FACS) plots (E) and frequencies (F) of IL‐22^+^ ILC3s in the intestinal lamina propria under different treatments (*n* = 5 per group). (G) Immunofluorescence analysis of Rorγt and IL‐22 expression in the intestine of mice with indicated treatments. (H) Serum IL‐22 levels in patients (*n* = 52 per group). *p* values were determined by the Student's *t*‐test (F) and Mann–Whitney *U* test (H).

We conducted a functional analysis of the intestinal RNA sequencing data to thoroughly investigate the mechanisms underlying gut barrier damage and increased susceptibility to TPN‐induced infection. Our findings indicate that the two most prominent Gene Ontology (GO) pathways identified are related to immune response and inflammatory response, as illustrated in Figure 2A. Kyoto Encyclopedia of Genes and Genomes (KEGG) pathway analysis and Gene Set Enrichment Analysis revealed significant modifications in the intestinal innate immune system (*p*_adj_ = 0.040, Figure 2B,C). We further analyzed the top 10 candidate cytokines based on RNA sequencing data to explore how TPN treatment might influence the immune system in organisms and found that the expression level of IL‐22 in TPN mice was significantly decreased (Figure 2D, Figure S3A). The IL‐22 is produced by ILC3s and other types of cells, including γδT, helper T17 (Th17), and Th22 cells [21, 22, 23]. However, in the intestines of both groups of mice, we observed no significant differences in the abundance of Th17 (*p* = 0.2035, Figure S3C,D), Th22 (*p* = 0.0941, Figure S3E,F), and γδT cells (*p* = 0.1233, Figure S3G,H), while ILC3s showed significant changes (*p* < 0.0001, Figure S4A–E). These alterations in ILC3s induced by TPN may contribute to the development of intestinal barrier damage and inflammation. We further evaluated the changes in the capacity of ILC3s to produce IL‐22 in both groups of mice. The results showed a significant decrease in the frequencies and number of IL‐22^+^ ILC3s due to TPN (*p* < 0.0001, Figure 2E,F), while the quantity and proportion of ILCs were not affected (Figure S4B,C). Furthermore, immunofluorescence analysis revealed a decrease trend in the IL‐22 and Rorγt expression levels in TPN‐treated mice's intestines (Figure 2G). Further analysis revealed reduced IL‐22 expression in the serum of TPN‐treated mice (Figure S4F). Consistent findings were observed in patients receiving H‐PN (Figure 2H).

**Figure 3:**
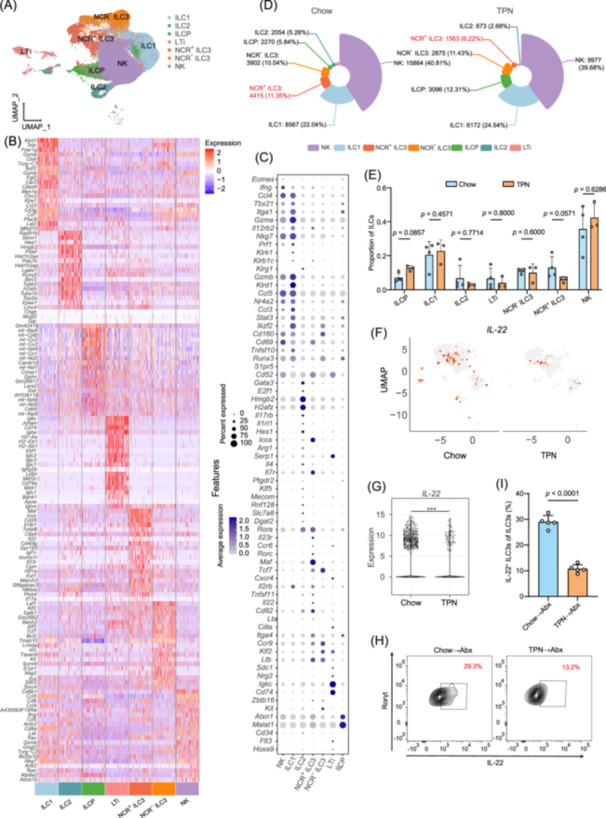
Single‐cell RNA sequencing (scRNA‐Seq) of ILCs from Chow and total parenteral nutrition (TPN) groups. (A) UMAP visualization of total ILC subsets across all locations. (B) Heatmap of the top 20 signature genes indicative of cell types. (C) Representative DEGs in cell clusters. The size of the dot indicates the percentage of cells within a cluster expressing the gene, while the color represents the average relative expression level of the gene. (D) Cell type percentages in ILCs from the Chow (*n* = 4) and TPN (*n* = 3) groups. (E) Cell type percentages from each sample in the Chow (*n* = 4) and TPN (*n* = 3) groups. (F) UMAP of NCR^+^ ILC3s shows higher *IL‐22* gene expression levels in the Chow group compared to the TPN group. (G) Violin plots displaying *IL‐22* expression in NCR^+^ ILC3s. Representative FACS plots (H) and percentages (I) of IL‐22^+^ ILC3s in the intestinal lamina propria following different treatments (*n* = 5 mice per group). ***, *p* < 0.001. *p* values were determined by the Man–Whitney *U* test (E) and the Student's *t*‐test (I). ILC3s, intestinal Group 3 innate lymphoid cells.

We isolated intestinal ILCs and conducted single‐cell sequencing analysis to investigate the specific variations in ILC3s further. The intestinal ILCs were categorized into seven cell subgroups (NCR^+^ ILC3, NCR^−^ ILC3, ILC1/2, NK, LTi, and ILCP) (Figure 3A). We identified the top 20 expressed genes and the characteristic markers of each cell subgroup. Specifically, *Tbx21* and *Itga1* are uniquely expressed by NK cells and ILC1, *Gata3* and *E2f1* are distinctive markers for ILC2, and *Rorc*, *Ccr6*, and *Maf* are characteristic genes for NCR^+^ ILC3s (Figure 3B,C). We observed the decrease in the proportion of NCR^+^ ILC3s in the TPN group (Figure 3D,E). Meanwhile, we found that the expression level of IL‐22 decreased correspondingly (Figure 3F,G), suggesting that the IL‐22 secreted by NCR^+^ ILC3s may play a protective role in the intestinal barrier [24]. We conducted an in‐depth analysis of the alterations in ILC3s populations within the mouse intestines following FMT. Our findings revealed a significant reduction in the proportion of IL‐22^+^ ILC3s (*p* < 0.0001, Figure 3H,I) and IL‐22 level (*p* < 0.05, Figure S4G–J) post‐transplantation with fecal matter from TPN‐treated mice. These results suggest that TPN modulates the gut microbiota, leading to a diminished ILC3s response within the intestinal immune microenvironment. This modulation likely contributes to compromised intestinal barrier integrity and an increased susceptibility to infection.

### TPN reduces *L. murinus* in the gut

**Figure 4:**
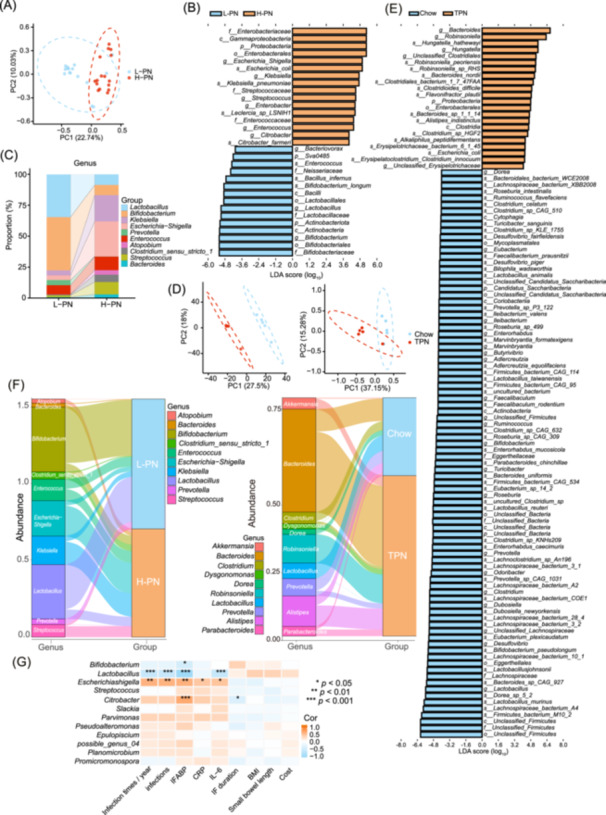
TPN induces gut dysbiosis in humans and mice. (A) PCoA for 16S rRNA gene sequences from fecal samples of patients (*n* = 16 individuals per group). (B) Significant microbial abundances in patients identified using linear discriminant analysis (LDA). (C) Top 10 abundant genera in the analysis of (B). (D) PCoA plots showing relative abundances at the genus level (left) or species level (right) in mice (*n* = 5 per group). (E) LDA analysis of abundant genera in mice. (F) Sankey diagram showing the top 10 abundant genera in both human and mice samples. (G) Heatmap illustrating the correlation between species abundance in the gut microbiota and clinical characteristics of patients with CIF. CIF, chronic intestinal failure. TPN, total parenteral nutrition.

TPN‐induced gut microbiota changes were analyzed by sequencing 16S ribosomal RNA (16S rRNA) from patient fecal samples to investigate the effective species of microbiota further (Figure S5A). Despite no significant differences in Alpha (α) diversity between the two groups of patients (Figure S5B), the principal coordinate analysis revealed that TPN significantly altered the gut microbiota of patients (Figure 4A). LDA effect size was used to analyze the bacterial composition, revealing that at the genus level, 10 genera contributed significantly to the differences in gut microbiota between the two groups (Figure 4B,C).

Metagenomic sequencing in a mouse model revealed significant differences in microbiota distribution between the two groups (Figure 4D, Figure S5C, *p* < 0.05). The bar graph of the linear discriminant analysis (LDA) value distribution illustrates that, at the genus level, 19 genera are characteristic differential taxa in the Chow group, while five genera are distinctive taxa in the TPN group. Notably, *Lactobacillus* genera exhibit higher LDA values (Figure 4E, Figure S5D,E).

Furthermore, a Sankey diagram was created based on the top 10 genera at the genus level in each sample or group. The findings revealed a concurrent reduction of *Lactobacillus* in both patients receiving H‐PN and in the TPN mouse model group (Figure 4F, Figure S5F–H). Based on a correlation analysis between the abundance of various bacterial genera and clinical characteristics of the patients, *Lactobacillus* showed a significant association (*p* < 0.001, Figure 4G). Thus, reducing *Lactobacillus* may be a significant factor in the TPN‐induced infection. Metagenomic analysis in mice showed strain‐level differences in four strains belonging to *Lactobacillus*: murinus, reuteri, johnsonii, and animalis (Figure 4E, Figure S5I, *p* < 0.05).

### Metabolically active *L. murinus* can ameliorate TPN‐related infection by regulating ILC3s

**Figure 5:**
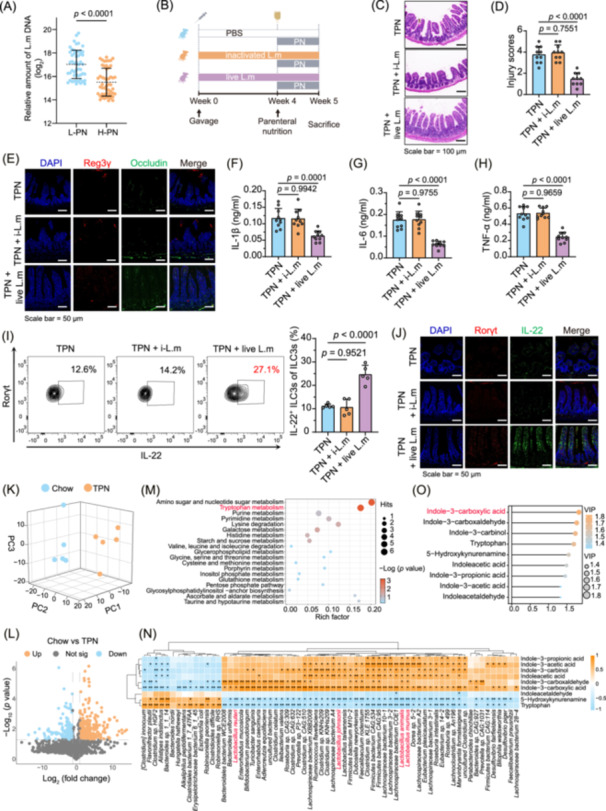
Metabolically active *L. murinus* ameliorate intestinal barrier damage by boosting intestinal Group 3 innate lymphoid cells (ILC3s). (A) Real‐time PCR (RT‐PCR) was conducted to quantify the abundance of *L. murinus* in feces from l‐PN and H‐PN patients (*n* = 52 individuals per group). (B) Experimental design scheme for live or inactivated *L. murinus* intervention. Representative intestinal HE staining (C) and injury scores (D) (*n* = 10 mice per group). (E) Immunofluorescence analysis of Reg3γ and Occludin expression in the intestine of mice with different treatments. Serum levels of IL‐1β (F), IL‐6 (G), and TNF‐α (H) of total parenteral nutrition (TPN) mice with different treatments (*n* = 10 per group). (I) Representative FACS plots and the percentages of IL‐22^+^ ILC3s in intestinal lamina propria after different treatments (*n* = 5 per group). (J) Immunofluorescence analysis of Rorγt and IL‐22 expression in the intestine of TPN mice after different treatments. The 3D principal component analysis (3D‐PCA) (K) and volcano plot (L) analyses of the distinct metabolic profiles from the feces of mice in the Chow versus TPN groups. (M) The metabolome‐wide pathways were enriched based on the 218 differential metabolites obtained from fecal content from Chow and TPN group mice (*n* = 5 per group). (N) Heatmap depicting the correlation between the abundance of gut microbiota at the species level and 9 differential tryptophan metabolites enriched in the Chow group mice. (O) Variable importance projection (VIP) scores according to 3D‐PCA show the contribution of metabolites to the classification of samples. A taxon with VIP score of > 1.5 was deemed significant in the discrimination process. *p* values were determined by Kruskal–Wallis *H* test with correction by Dunnett's *t*‐test (A) and one‐way ANOVA (post hoc analysis used Dunnett's *t*‐test; D and F–I). All statistical tests were two‐sided.

We conducted a strain intervention experiment on TPN mice to investigate the biological functions of Lactobacillus in TPN‐induced infection (Figure S6A). We found that *L. murinus* significantly improved intestinal barrier integrity (Figure S6B–D). Subsequently, fecal samples from patients showed that *L. murinus* abundance was significantly reduced in H‐PN group feces (*p* < 0.0001, Figure 5A). We further analyzed the clinical characteristics of CIF patients in relation to *L. murinus* and identified a significant negative correlation between the abundance of *L. murinus* and the occurrence of infections in these patients (*p* = 0.0017, Figure S6E). These results demonstrate the association between *L. murinus* reduction and TPN‐related intestinal barrier damage and infection in clinical settings.

We administered oral gavage TPN mice with phosphate‐buffered saline (PBS), inactivated *L. murinus* (i‐L. m), or live *L. murinus* (live L. m) to further investigate the role of *L. murinus* (Figure 5B). We found that only live *L. murinus* ameliorated intestinal pathology (Figure 5C,D, Figure S6F), and concurrently upregulated markers such as ZO‐1, Occludin, Reg3γ, and lysozyme (Figure 5E, Figure S6G,H). Furthermore, live *L. murinus* significantly reduced the levels of inflammatory factors (*p* < 0.0001, Figure 5F–H), indicating the protective role of metabolically active *L. murinus*.

Further analysis of IL‐22 expression levels and the abundance of *L. murinus* in patients revealed a positive correlation (*rs* = 0.689, *p* < 0.001, Figure S6I). Post‐intervention with live *L. murinus*, there was a significant increase in the frequencies of IL‐22^+^ ILC3s (*p* < 0.0001, Figure 5I). Immunofluorescence assays further demonstrated that live *L. murinus* upregulated the expression of intestinal Rorγt and IL‐22 (Figure 5J). These findings suggest that live *L. murinus* can enhance the differentiation of IL‐22^+^ ILC3s, thereby improving intestinal barrier function and resistance to infection.

### ICA is critical for *L. murinus* to ameliorate TPN‐related intestinal barrier damage

We analyzed the feces of TPN‐treated mice using liquid chromatography‐mass spectrometry (LC‐MS) to investigate their metabolite composition. The 3D principal component analysis (3D‐PCA) and volcano plot analysis highlighted distinct metabolic profiles between the TPN and Chow groups, revealing 218 metabolites (Chow vs. TPN, up 117 and down 101) as differential metabolites (Figure 5K,L). We conducted a KEGG pathway analysis using these 218 differential metabolites. The KEGG pathway analysis identified 19 enriched metabolic pathways, with the top five being aminoglycoside and nucleoside sugar metabolism, tryptophan metabolism, histidine metabolism, glycerophospholipid metabolism, and fructose and mannose metabolism (*p* < 0.001, Figure 5M). Among these, tryptophan metabolism exhibited the most difference, with a *p*‐value = 0.0009.

The tryptophan metabolic pathway is critical for mediating the gut microbiota's influence on host metabolism and immune homeostasis. Correlation analysis between bacterial strains and tryptophan metabolites revealed a strong positive relationship between indole compounds, such as ICA, indole‐3‐acetylaldehyde, and indole‐3‐propionic acid, and the *L. murinus* genus (*p* < 0.05, Figure 5N). Notably, ICA demonstrated the highest variable importance in the projection (VIP) score (Figure 5O), with a significant positive correlation between ICA levels and *L. murinus* abundance in the feces (*rs* = 0.721, *p* < 0.001, Figure S6J). In the feces of patients receiving H‐PN, the ICA levels decreased (Figure S6K). Similarly, following intervention with live *L. murinus*, it was observed that the level of ICA significantly increased in the feces of TPN mice (*p *< 0.0001, Figure S6L). Thus, *L. murinus* may protect the intestinal barrier through ICA in both TPN mice and patients.

**Figure 6:**
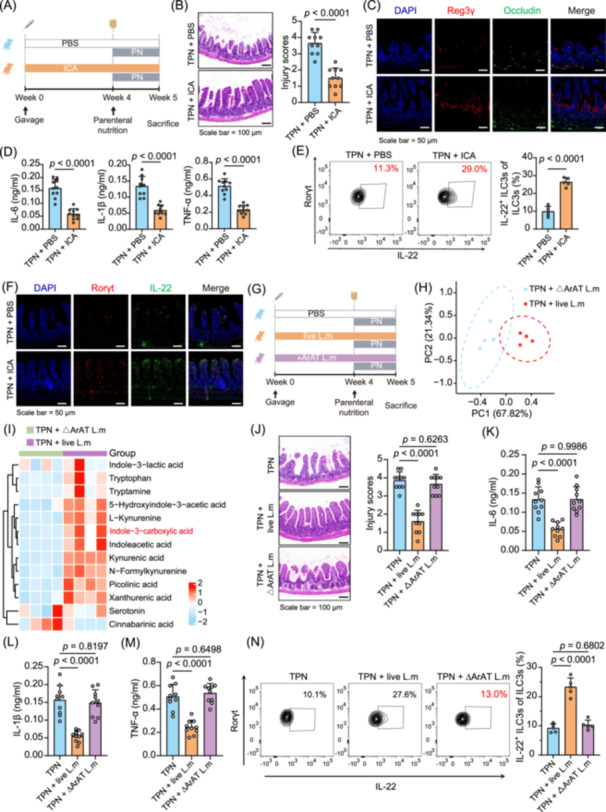
Indole‐3‐carboxylic acid (ICA) is a critical metabolite responsible for modulating intestinal Group 3 innate lymphoid cells (ILC3s). (A) Experimental design scheme for ICA intervention. (B) Representative mouse intestinal HE staining and injury scores (*n* = 10 per group). (C) Immunofluorescence analysis of Reg3γ and Occludin expression in the intestine of TPN mice treated with PBS or ICA. (D) Serum levels of IL‐1β, IL‐6, and TNF‐α in TPN mice with different treatments (*n* = 10 per group). (E) Representative FACS plots and the percentages of IL‐22^+^ ILC3s in intestinal lamina propria after different treatments (*n* = 5 mice per group). (F) Immunofluorescence analysis of Rorγt and IL‐22 expression in the intestines of TPN mice after different treatments. (G) Experimental design scheme for live or ∆ArAT *L. murinus* intervention. (H) PLS‐DA for the profiles of metabolite in feces from TPN mice receiving ΔArAT or live *L. murinus* (*n* = 4 per group). (I) Heat map of tryptophan‐targeted metabolomics in fecal samples from TPN mice that received either ΔArAT or live *L. murinus* (*n* = 4 per group). (J) Representative mouse intestinal HE staining and injury scores (*n* = 10 per group). Serum levels of IL‐1β (K), IL‐6 (L), and TNF‐α (M) of TPN mice with different treatments (*n* = 10 per group). (N) Representative FACS plots and the percentages of IL‐22^+^ ILC3s in intestinal lamina propria after different treatments (*n* = 5 per group). *p* values were determined by the Student's *t*‐test (B and D, E) and one‐way ANOVA (post‐hoc analysis used Dunnett's t‐test; J–N).

We supplemented ICA and PBS to TPN mice to test this hypothesis (Figure 6A). Gavage administration of ICA resulted in fecal ICA concentrations reaching levels similar to those of live *L. murinus* (Figure S7A, Figure S6L). Notably, ICA treatment improved intestinal pathology (Figure 6B, Figure S7B,C) and concurrently upregulated markers such as ZO‐1, Occludin, Reg3γ, and lysozyme (Figure 6C, Figure S7D,E). Additionally, treatment with ICA significantly reduced the levels of inflammatory factors (*p* < 0.0001, Figure 6D). After intervention with ICA, the frequencies of IL‐22^+^ ILC3s significantly increased (*p* < 0.0001, Figure 6E), leading to an upregulation of intestinal IL‐22 expression levels (Figure 6F).

Aromatic amino acid aminotransferase (ArAT) is required for the production of ICA in *L. murinus* (Figure S7F). Subsequently, to determine whether depletion of ArAT activity would eliminate the protective effect of *L. murinus* on the intestinal barrier, a mutant strain without functional ArAT was constructed (Figure S7G,H). We performed LC‐MS analysis on fecal samples from TPN mice infused with *L. murinus* ΔArAT (Figure 6G), and Orthogonal Partial Least Squares‐Discriminant Analysis revealed distinct metabolic profiles following interventions with wild‐type and mutant *L. murinus* (Figure 6H). A significant reduction in ICA levels in the feces of TPN mice was observed following ArAT depletion (Figure 6I); however, no improvement was noted in the intestinal pathology or markers associated with the intestinal barrier in these mice (Figure 6J, Figure S7I–L). Additionally, the expression of inflammatory markers (*p* > 0.01, Figure 6K–M) and the percentage of IL‐22^+^ ILC3s (*p* = 0.6802, Figure 6N) exhibited no significant changes, indicating that the beneficial effects of *L. murinus* were abrogated following ΔArAT knockout. In summary, ArAT and its product ICA are essential for *L. murinus* in mitigating TPN‐associated infection.

### ICA promotes ILC3s to express IL‐22 by targeting Rorγt

To further investigate whether *L. murinus* and ICA exert their effects through the regulation of ILC3s, we generated Rorγt knockout (*Rorc* ^−/−^) mice to deplete ILC3s. TPN modeling and intervention were subsequently conducted on *Rorc* ^−/−^ mice (Figure S8A). The results revealed that the beneficial effects of *L. murinus* and its metabolite ICA on the intestinal barrier were inhibited (Figure S8B–D), indicating that loss of ILC3s impedes the protective role of *L. murinus* and ICA.

**Figure 7:**
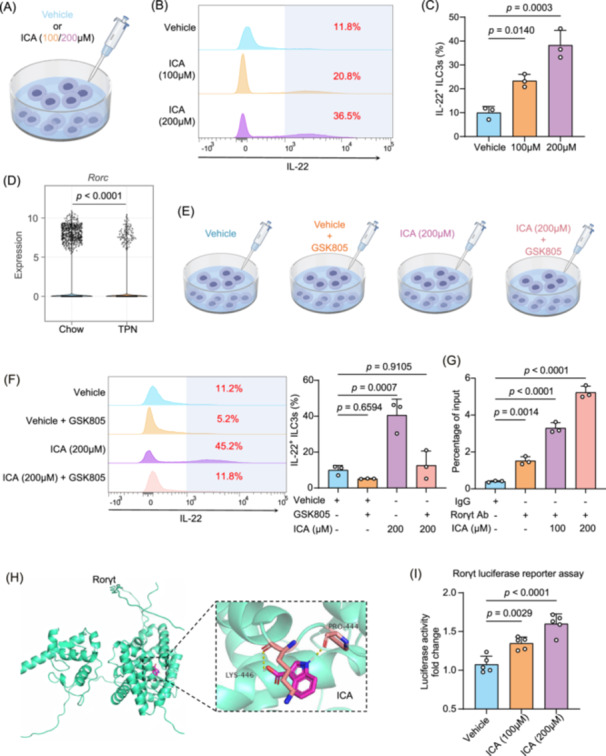
ICA promotes the function of intestinal Group 3 innate lymphoid cells (ILC3s) by targeting Rorγt. (A) ILC3s treated with varying concentrations of indole‐3‐carboxylic acid (ICA). Representative FACS plots (B) and frequencies (C) of IL‐22^+^ ILC3s after treatment with various concentrations of ICA for 72 h. (D) Violin plots displaying *Rorc* expression in NCR^+^ ILC3s. (E) ILC3s treated with ICA (200 µM), GSK805 (0.5 µM), or a combination of both. (F) Representative FACS plots and the frequencies of IL‐22^+^ ILC3s following different treatment. (G) ChIP‐qPCR analysis of the effect of ICA on Rorγt binding to the IL‐22 gene promoter (*n* = 3). (H) Identification of ICA as a potential Rorγt agonist through molecular docking, demonstrating hydrophobic van der Waals interactions (carbon atoms in red) within the Rorγt‐binding pocket. (I) Luciferase reporter assay demonstrating that ICA activates Rorγt transcriptional activity. A PGL3 construct containing the Rorγt‐binding sequence was transfected into HEK‐293 cells. The cells were then treated with vehicle (DMSO) or indicated concentrations of ICA followed by luciferase activity assay. Date are presented as mean ± SEM (*n* = 5). *p* values were determined by one‐way ANOVA (post‐hoc analysis used Dunnett's *t*‐test; C, F, G, and I). All statistical tests were two‐sided. ChIP‐qPCR, chromatin immunoprecipitation‐quantitative polymerase chain reaction.

Next, to explore how ICA enhances ILC3s function, we isolated ILC3s from the intestines of mice and conducted in vitro intervention experiments (Figure 7A). Compared with the vehicle group, the frequencies of IL‐22^+^ ILC3s increased when treated with ICA (Figure 7B,C). Through differential gene analysis of NCR^+^ ILC3s from the data of scRNA‐seq [25, 26], we identified the genes that might be involved in the regulation of IL‐22 secretion by ILC3s. Among these, we observed that *Rorc* expression in the TPN group significantly decreased (*p* < 0.0001, Figure 7D). In contrast, expression levels of the aryl hydrocarbon receptor (*Ahr*), another key transcription factor potentially involved in the production of IL‐22 by ILC3s, exhibited similar expression levels in NCR^+^ ILC3s from both Chow and TPN groups of mice (Figure S8E). This finding suggests that ICA may regulate ILC3s function through *Rorc*. Consistently, both in vitro and in vivo experiments demonstrated that ICA elevates *Rorc* mRNA and protein levels (Figure S8F–I). We then compared the effects of ICA on IL‐22^+^ ILC3s in the presence of GSK805 (0.5 µM), a Rorγt inhibitor (Figure 7E), and found that GSK805 significantly blocked ICA‐induced differentiation of IL‐22^+^ ILC3s (Figure 7F).

We next assessed whether ICA affects Rorγt binding to its target genomic elements. Using chromatin immunoprecipitation followed by quantitative polymerase chain reaction (ChIP‐qPCR), we evaluated Rorγt occupancy at the *IL‐22* promoter region and found that ICA significantly enhanced the binding of Rorγt to this site (Figure 7G, Figure S8J). Molecular docking simulations revealed that ICA may form a stable complex with Rorγt, with a binding energy of −6.1 kcal/mol (Figure 7H). Luciferase reporter gene assays further revealed that ICA markedly activated Rorγt transactivation activity (Figure 7I). This result suggests that ICA may enhance Rorγt transcriptional activity and promote IL‐22 expression (Figure S8K).

## DISCUSSION

TPN administration may damage the intestinal barrier, promoting bacterial and endotoxin translocation into the systemic circulation, consequently inducing infections and exacerbating adverse clinical outcomes in patients [3, 27, 28]. Preventing TPN‐associated intestinal barrier damage and reducing TPN‐related infections are pivotal in treating patients with CIF [29]. Although TPN reportedly induces dysregulation of the gut microbiota [3, 30], the specific mechanisms by which TPN‐induced microbiota dysregulation contributes to intestinal barrier damage and subsequent infection remain largely unexplored.

In present study, we observed that TPN inhibited intestinal innate immune function in mice. Furthermore, through flow cytometry and scRNA‐seq analyses, we identified a reduction in both the number and proportion of intestinal IL‐22^+^ ILC3s as a consequence of TPN administration. Numerous studies have demonstrated ILC3's protective effects in the intestine [31, 32, 33]. These cells can integrate cytokine‐mediated information and transmit it to IECs and resident immune cells to enhance intestinal immune and metabolic homeostasis [18]. A previous study indicated that the cytokines generated by activated ILC3s, specifically IL‐22, stimulate antimicrobial peptide production (e.g., Reg3γ) and mucins in IECs, fortifying the gut barrier's function [34]. We hypothesized that the attenuation of ILC3s function would be intricately linked to TPN‐associated infections. However, the precise role of ILC3s in the context of TPN‐associated infections remains to be elucidated.

Further research demonstrated that TPN‐induced dysbiosis mediates alterations in ILC3s function and is associated with TPN‐related infections. Transplantation of fecal microbiota from donors in the TPN group resulted in a reduced proportion of IL‐22^+^ ILC3s in the intestines of ABX mice, thereby increasing their susceptibility to intestinal barrier damage and infection. Conversely, transplantation of fecal microbiota from donors in the Chow group did not induce these alterations. Furthermore, a decrease in *L. murinus* was observed in fecal samples from both clinical patients undergoing TPN and TPN‐treated mice, suggesting that *L. murinus* may play a critical role in modulating ILC3s function and is implicated in TPN‐associated infections. To our knowledge, no studies have shown that *L. murinus* can regulate ILC3s and improve TPN‐related infections. Other studies have also observed that there is an interaction between the host and the microbial community. The researchers identified a decrease in the abundance of *Parabacteroides goldsteinii* in the intestine under aspirin treatment, inhibiting its bile acid metabolism and causing damage to the intestinal barrier [15].

*L. murinus* can modulate tryptophan metabolism and exert protective effects in various diseases [35, 36, 37, 38]. Its beneficial effects can be attributed to its antibacterial activity and immunomodulatory function. *L. murinus* can prevent salt‐sensitive hypertension by inhibiting the differentiation of Th17 cells through indole lactic acid production [36]. Triggering TLR2 also induces macrophages to release IL‐10 to heal intestinal I/R injuries [37]. Recent studies have indicated a positive correlation between *L. murinus* and intestinal barrier function [39, 40, 41]. Moreover, *L. murinus* modulates macrophage polarization and ameliorates chemically induced intestinal inflammation through extracellular vesicle secretion [42]. These studies have motivated us to investigate how *L. murinus* improves ILC3s and TPN‐associated infection.

Our study demonstrates that ICA is a crucial molecule produced by *L. murinus* that improves the function of ILC3s, with no prior research reported in this context. Yu et al. have indicated that ICA produced by *Lactobacillus helveticus* enhances CD8^+^ T cell functions by inhibiting the differentiation of CD4^+^ Treg cells, improving anti‐PD1 therapeutic efficacy in colorectal cancer [43]. Given that previous studies have only reported the regulatory role of ICA on the immune function of plants [44, 45], this study, along with Yu et al.'s study [43], highlights the potential application prospects of ICA in immune regulation for diseases in animals or human.

We found that *L. murinus* and its derivative ICA can directly target Rorγt to promote ILC3s secretion of IL‐22, ameliorating intestinal barrier damage and thereby reducing TPN‐induced infection. Previous studies have characterized gut microbiota derivatives as functioning both as partial *Ahr* agonists and competitive *Ahr* inhibitors, regulating *Ahr* activation to affect the functionality of immune cells [46, 47, 48]. However, our study suggests a novel mechanism by which the gut microbiota and its derivatives directly regulate immune cells to protect the intestinal barrier, reducing infection.

Our study has several limitations. The mechanisms by which ILC3s improve TPN‐associated intestinal barrier damage require further exploration. Current evidence suggests that ILC3s enhance intestinal immunity and alleviate inflammation through various mechanisms [49, 50], such as protecting intestinal stem cells from genetic stress [21, 51, 52], secreting granulocyte‐macrophage colony‐stimulating factor or IL‐2, and modulating T cells and macrophages [53, 54]. However, a comprehensive understanding of how ILC3s specifically mitigate TPN‐induced intestinal barrier damage remains to be elucidated.

Additionally, our study observed significant alterations in cytokines, such as IL‐1β, IL‐5, IL‐27, and IL‐17A, induced by TPN. These changes underscore the complex regulatory network influencing intestinal immune function during TPN. While our research focused on IL‐22‐producing immune cells, we did not investigate the contributions of other cytokine‐producing cells, such as IL‐1β‐producing macrophages and neutrophils [55, 56], or IL‐5‐producing eosinophils and mast cells [57]. These interactions and their impacts on intestinal barrier function will be systematically explored in future studies.

Finally, our inability to employ the STOP/CD4 mouse model for targeted interventions in ILC3s represents a limitation of our study. Addressing this gap in future research will provide a more precise understanding of ILC3‐specific mechanisms and their therapeutic potential in managing TPN‐related intestinal barrier dysfunction.

## CONCLUSION

In conclusion, our study confirmed that *L. murinus* and its tryptophan catabolite, ICA, can facilitate the differentiation of IL‐22^+^ ILC3s, thereby protecting intestinal barriers and reducing TPN‐associated infection. Furthermore, we demonstrated that ICA enhances IL‐22 secretion by ILC3s through the activation of Rorγt, thereby mitigating TPN‐induced intestinal barrier damage. These findings elucidate a novel mechanism of host‐microbiota metabolism‐immune regulation and underscore the potential of *L. murinus* or ICA as adjunct therapies to alleviate TPN‐associated infection in patients with CIF.

## METHODS

### Study participants, specimen gathering

A total of 338 participants were enrolled in the study (Figure S1) between August 2017 and August 2022. The inclusion criteria were as follows: (1) age of 18–75 years; (2) expected PN duration > 14 days; (3) stable medical condition; and (4) CIF diagnosis. The exclusion criteria were: (1) antibiotic use within a month before or after admission; (2) severe liver and kidney failure; (3) pregnancy; and (4) major alterations to the PN regimen. The informed consent was signed by each participant before the collection of clinical data, fecal samples, and blood samples, and each participant was compensated. The study received approval from the Ethics Committee of Jinling Hospital (No. 2021NZKY‐024‐01).

### Parenteral nutrition strategy

During hospitalization, all participants were given individualized nutrition support therapy. Each day, the personalized dietary plans were modified according to resting energy use, nitrogen levels, blood nutrition indicators, and fluid balance throughout the hospital stay.

### Animal studies

C57BL/6 and *Rorc* ^−/−^ mice were obtained from GemPharmatech Co., Ltd (Nanjing, China). This study utilized 12‐week‐old male C57BL/6 mice maintained under specific pathogen‐free conditions. These mice were housed under 20°C–26°C conditions, a 12‐h light and dark cycle, and 40% to 70% humidity. Before the study, all mice were given food and water, and their activities were not restricted. We calculated the sample size for each group of mice to be 10, using Powerandsamplesize (https://powerandsamplesize.com/).

### TPN mouse model

The molding method is consistent with descriptions in previous research reports [8]. Twenty mice were assigned to two groups using a completely randomized approach based on the treatment received, with 10 mice in each group. In both experimental groups, mice were allowed to drink water on the 1st day after surgery and, then, received either standard feed (Chow group) or TPN at a rate of 0.40 mL/h for 7 days, after which they were killed.

### FMT mouse model

The study involved two groups of 12‐week‐old male C57BL/6 mice, maintained under specific pathogen‐free (SPF) conditions: Chow→Abx (*n* = 10) and TPN→Abx (*n* = 10). Both groups were fed sterile, 60Co‐irradiated food to ensure consistent dietary and microbial exposure (1010085; Xietong) [17]. The mice were initially provided with a mixture of antibiotics in drinking water for 2 weeks, consisting of ampicillin, metronidazole, and neomycin (1 g/L), vancomycin (500 mg/L), and sucralose (8 g/L) [58]. Then, the mice were gavaged every other day for 4 weeks with diluted cecal contents collected from the Chow or TPN groups. The cecal contents were weighed, pooled, and mixed with a pre‐reduced 30% glycerol/PBS solution to achieve a final concentration of 100 mg/mL. Then, the mixture was filtered through a 100‐µm nylon filter in an anaerobic chamber to remove fibrous and large particulate matter. For each gavage, 0.25 mL of the diluted cecal contents was aliquoted and stored at −80°C until use.

### ICA oral mouse model

A total of 20 mice were assigned randomly to two groups (each group *n* = 10): TPN + PBS and TPN + ICA. At 4 weeks before the TPN, ICA was administered at a dose of 10 mg/kg of body weight via oral gavage three times a week until 1 week after TPN, after which the mice were killed. The dosage of ICA is based on previous studies [43]. In the TPN + PBS group, mice were gavaged with a vehicle control consisting of PBS.

### Single‐strain intervention

Thirty mice were randomly assigned to three groups (each group *n* = 10): TPN + PBS, TPN + inactived *L. murinus*, and TPN + live *L. murinus*. Three groups of 30 mice were randomly selected (each group *n* = 10) for the ΔArAT *L. murinus* intervention experiment: TPN + PBS, TPN + live *L. murinus*, and TPN + live ΔArAT *L. murinus*. The ΔArAT *L. murinus* was purchased from Biosafe Plasmid Bacterial Strain Resource Company. In MRS Broth (DF0881‐17‐5; BD Difco), *L. murinus* (JCM1717; ATCC), *Lactobacillus reuteri* (23272, ATCC), *Lactobacillus johnsonii* (33200, ATCC), *Lactobacillus animalis* (JCM 5670, ATCC), and ΔArAT *L. murinus* were cultured anaerobically at 37°C. The inactivated *L. murinus* was incubated for 15 min at 65°C. Beginning 4 weeks before the administration of TPN and continuing until the sacrifice of the mice, they were gavaged daily with 10^8 colony‐forming units in 200 μL of PBS.

### Ussing chamber study

Ileums were taken from killed animals, opened, and washed three times in cold Krebs's solution (2.5 mmol/L CaCl_2_, 1.98 g/L glucose, 4.7 mmol/L KCl, 1.2 mmol/L NaH_2_PO_4_, 117 mmol/L NaCl, 25 mmol/L NaHCO_3_, and 1.2 mmol/L MgCl_2_). The tissue portions were mounted and tested in Ussing chambers (Physiologic Instruments) with an exposed surface area of 0.03 cm^2^.

### Isolation of intestinal laminapropria cells

The small intestines were isolated and longitudinally incised [59]. Fecal matter was rinsed away using saline solution, and adipose tissue, Peyer's patches, and mesentery were excised using ophthalmic forceps and shears. We then separated the intestines into 1‐cm segments and washed them with PBS. Subsequently, the intestinal segments were transferred to a container containing a washing solution consisting of HEPES buffer (10 mM), DTT (1 mM), and EDTA (30 mM). The segments were agitated at 200 rpm at 37°C for two washes, each lasting 20 min. The remaining tissues were transferred into C tubes with a digestive enzyme solution and incubated at 37°C with agitation at 150 rpm for an additional 20 min. The C tubes were placed on a gentleMACS^TM^ Dissociator (130‐093‐235, Miltenyi Biotec) to obtain a cell suspension. Cells were mixed with peroll solution and subjected to density gradient centrifugation. Subsequently, the intermediate cell layer was reconstituted in DMEM.

### Flow cytometry

Monoclonal antibodies conjugated to fluorescently labeled dyes were used to stain single‐cell suspensions. Before staining the cell surface, Fixable Viability Dye (65‐0865‐14, eBioscience) was used to detect live/dead cells. Mouse antibodies were used to stain surface markers as the following: CD90.2 (53‐2.1), CD45 (30‐F11), lineage markers (17A2/RB6‐8C5/B220/Ter‐119/M1/70), NK1.1 (PK136), and KLRG1 (2F1). All antibodies listed above were used according to the manufacturer's instructions.

Cells were extracted ex vivo and exposed to 20 ng/mL IL‐23 (200‐23, PeproTech) for 4 h before testing. After surface staining with the antibodies mentioned above, the targeted cells were fixed and permeabilized by Foxp3/Transcription Staining Buffer (00‐5523‐00, eBioscience). The cells were then stained with IL‐22‐PerCP eFluor710 (IC582N, R&D) and ROR gamma (t) (12‐6981‐82, Invitrogen). FlowJo version 10.8.1 software (Tree Star) was used to analyze stained cells acquired with an Attune NxT flow cytometer (BD Biosciences). As illustrated in Figure S4A, FACS gating was employed to identify ILC3s.

### Isolation and culture of ILC3s

A SORP FACSARIA II flow cytometer selected Lineage^−^CD45^+^CD90.2^+^NK1.1^−^KLRG1^−^ cells (ILC3s) with 97% purity. Afterwards, RPMI‐1640 medium was added to 96‐well plates with 10,000 cells cultured per well for 12 h at 37°C. A 10% fetal bovine serum (FBS) medium was used with 2‐mercaptoethanol (80 μM), Sodium Pyruvate (1 mM), Non‐essential amino acids, glutamine (2 mM), HEPES buffer (10 mM), 100 µg/mL streptomycin, and 100 units/mL penicillin, all sourced from Gibco. Treatment was conducted with mouse recombinant IL‐23 at 40 ng/mL (200‐23; PeproTech), and ICA at 100 and 200 μM concentrations. After 72 h, IL‐22 was detected in supernatants, and mRNA and flow cytometry analyses were conducted following cell harvest.

### ChIP‐qPCR

ChIP assay was conducted using a commercial enzymatic ChIP kit (53040; Active Motif). ILC3 nuclear lysates, post 1% formaldehyde cross‐linking, were sonicated to fragment the chromatin, followed by immunoprecipitation with Rorγt (14‐6988‐82, eBioscience) antibody or control IgG. Amplification of target genomic regions from both input and immunoprecipitated samples was performed via both PCR and quantitative real‐time PCR (qRT‐PCR). The PCR products underwent electrophoresis on 2% agarose gels and were visualized with a BG‐gdsAUTO550 gel imaging system (Baygene), and then quantified using ImageJ. Primers used for the *IL‐22* promoter putative Rorγt binding motif were 5'‐CTAGTTGTCAGGTGCTATCT‐3' (sense) and 5'‐TGTGCAAGCATAAGTCTCA‐3' (antisense).

### Luciferase reporter assay

Luciferase reporter plasmids for *IL‐22* promoter, Rorγtp‐luc, containing the Rorγt‐binding sequence, were constructed by inserting the promoter sequences into the pGL3‐basic vectors (Promega). HEK‐293 cells were seeded in 12‐well plates with DMEM containing 1% FBS and no antibiotics. Upon reaching 50% confluence, the cells were transiently transfected with either Rorγtp‐luc or Rorγtcp‐HA, along with a Renilla luciferase reporter as internal control. After 24 h posttransfection, the cells were exposed to vehicle control, 100 μM ICA, or 200 μM ICA for 24 h. Then, the cells were harvested and lysed for luciferase assays. Luciferase activities were assayed using a dual luciferase reporter assay kit (DL101‐01; Vazyme) and evaluated by the Dual‐Glo® Luciferase Assay System (Promega). The results were normalized to renilla luciferase levels and expressed as relative fold changes.

### DNA extraction of *L. murinus*

A TIANamp Stool DNA Kit (4992205; Tiangen) was used to extract DNA from feces, following the manufacturer's instructions. iTaq Universal SYBR (Bio‐Rad; 1725125) was used for quantitative PCR (qPCR) using Fast real‐time PCR (RT‐PCR) System (Thermo Fisher Scientific; 7900HT). Primers of *L. murinus* are listed in Table S2.

### RNA extraction and RT‐PCR

TRIzol LS Reagent (15596018CN, Invitrogen) was used to extract total RNA from mouse intestine and cell lines. The GeneAmp PCR System 9700 (Applied Biosystems) was used to analyze the relative expression of mRNAs from the reverse‐transcribed of 1 μg total RNA with the PrimeScriptTM RT reagent Kit (RR037B, Takara). 2^–ΔCt^ was used to compare cycle threshold values (Ct values) obtained from the samples. β‐actin was used as an internal reference to detect host genes. Table S2 presents all primers.

### Western blot analysis

SDS‐PAGE (4% and 8%) was used for protein extraction from frozen small intestinal tissue. The samples were then transferred to polyvinylidene fluoride membranes for further analysis. Antibodies against ZO‐1 (ab59720, Abcam), Occludin (ab216327, Abcam), REG3G (ab239610, Abcam), Lysozyme (ab108508, Abcam, Cambridge, UK), and GAPDH (BA3874, BOSTER) were used following the manufacturer's instructions. Western blots were performed with the EPS600 electrophoresis apparatus (Tanon), viewed with Image Lab (version 4.0, Bio‐Rad), and analyzed semi‐quantitatively with ImageJ US NIH; 1.52a). Key resources used in the study are listed in Table S3.

### Immunofluorescence

For paraffin‐embedded slides, tissues were fixated overnight in 10% formalin, embedded in paraffin, and sliced into 5 mm thicknesses. After the paraffin was removed from the slides, they were rehydrated using alcohol and PBS solutions. A 0.2% Triton X‐100 solution (9036‐19‐5, Sigma‐Aldrich) was used for permeabilization, followed by 10% normal donkey serum (D9663, Sigma‐Aldrich) for blocking. On the slides, primary antibodies were incubated at 4°C overnight. Following the application of the primary antibodies, the slides were left at ambient temperature for an hour with the appropriate secondary antibodies. DAPI‐stained slides were mounted with ProLong^TM^ Gold Antifade reagent (Invitrogen) and examined under a Zeiss LSM880 confocal microscope.

### Molecular docking

The docking study of Rorγt and ICA was performed using AutoDock Vina [60]. The program utilizes the “iterated local search” algorithm to perform continuous local searches and identify the optimal molecular docking conformation. The solution with the lowest binding energy is selected as the final outcome.

### Hematoxylin and eosin (HE) staining and Chiu's score

Mouse intestines were fixed in stages, embedded in paraffin, sliced into sections, and placed on slides for HE staining. Blinded histological scoring was performed by two independent pathologists after staining, following previously described standard [15]. Statistical analysis was conducted for all individuals in each group based on the average values of one slice of intestinal tissue.

### 16S rRNA‐sequencing experiment

The sequencing of 16S rRNA was performed similarly to the protocol outlined in a prior study [8]. Subsequent data analyses were conducted utilizing the Shengxin Cloud tools, a complimentary online platform provided by Hangzhou Kaitai Biotechnology Co., Ltd. (Nanning, China; https://kaitai.cloud).

### mRNA‐sequencing

Tissue samples from the small intestine in Chow and TPN groups were collected, and total RNA was extracted using RNAiso Plus. The mRNA sequencing was on the NovaSeq™ X Plus, PE150 platform (Illumina). Clean sequencing data were aligned to the mouse genome (GRCm39) using software HISAT2 (V2.2.1, http://ccb.jhu.edu/software/hisat2) and annotated with the Ensembl database (Mus_musculus. GRCm39.109. gtf) [61]. Transcriptome assembly was conducted using the StringTie software package (https://ccb.jhu.edu/software/stringtie/). Quantitative analysis was performed using HTSeq (http://htseq.readthedocs.io/en/release_0.9.1) [62]. DEGs were identified using edgeR software (http://www.bioconductor.org/packages/release/bioc/html/edgeR.html) [63]. The threshold for differential gene selection is set at |log2FC| > 1 and *p*_adj_ < 0.05. We performed GO/KEGG/Reactome analysis was performed using the clusterProfiler software (http://www.bioconductor.org/packages/release/bioc/html/clusterProfiler.html) [64].

### Metagenomic sequencing experiment

Clean data from fecal samples were generated using Illumina HiSeq sequencing platforms and processed with Readfq V8. Host‐derived reads were filtered using Bowtie 2.2.4 software, which blasted clean data against the reference database (http://bowtiebio.sourceforge.net/bowtie2/index.shtml). A SOAPdenovo (V2.04) analysis is then performed on the clean data (http://soap.genomics.org.cn/soapdenovo.html). Subsequently, SOAPdenovo (V2.04) and MEGAHIT (V1.0.4) were employed for mixed assembly.

### Metagenomic sequencing statistical analysis

Based on the software MetaGeneMark (http://topaz.gatech.edu/GeneMark/, version 2.10), all five Scaftigs were predicted to contain open reading frames (ORFs). The software CD‐HIT (http://www.bioinformatics.org/cd-hit, V4.5.8) was employed to predict ORFs, eliminating redundancy and generating a unique initial gene catalog. Subsequently, an initial gene catalog was created based on clean data from each sample using Bowtie (Los Angeles; version 2.2.4), with the number of reads for each gene determined according to specific parameter settings. Subsequent analyses, including basic statistical summaries, correlation analyses, and Venn diagrams of gene counts, were conducted using abundance data from the gene catalog. The software DIAMOND (https://github.com/bbuchfink/diamond/, version 0.9.9) was employed to align the unigenes with sequences from bacteria, viruses, fungi, and archaea. Subsequent data analyses were conducted utilizing the Shengxin Cloud tools, a complimentary online platform provided by Hangzhou Kaitai Biotechnology Co., Ltd. (https://kaitai.cloud).

### Untargeted metabolomics

The Orbitrap LC‐MS mass spectrometer with SCIEX 6500 QTRAP + system was used to analyze metabolites in the cecal contents of mice. Ion source parameters settings were as follows: Curtain Gas: 25 psi, Temperature: 400°C, IonSpray Voltage: +5500/− 4500 V, Ion Source Gas 2: 60 psi, Ion Source Gas 1: 55 psi, and DP: ±100 V. Metabolite quantification was performed using the software Compound Discoverer (Thermo Fisher Scientific). Metabolites demonstrating significant differences were identified according to VIP scores exceeding 1.5, with the fold change being greater than 2.0 or less than 0.5 and the *p*‐value being less than 0.05. Enrichment analysis of the KEGG pathway for these metabolites was conducted using statistical methods. Data visualization was performed using R version 3.1.3 (R Software for Statistical Computing).

### Quantification of tryptophan by LC‐MS

Separation was executed using the Ultra High‐Performance Liquid Chromatography system (Agilent 1290 Infinity, Agilent). Standard samples were stored at 4°C in the automatic injector, and the column temperature was maintained at 40°C. A quality control (QC) sample was periodically introduced into the sample queue following a predetermined number of experimental samples to evaluate system stability and repeatability. A blend of standard substances corresponding to the targeted analytes was added to the sample queue to aid in the correction of chromatographic retention times. The mass spectrometric analysis was conducted utilizing the SCIEX 5500 QTRAP mass spectrometer. Multiquant 3.0.2 software was used to extract the chromatographic peak areas and retention times. The retention times were corrected by employing standard substances of the target compounds to facilitate the identification of metabolites.

### Experiment of single‐cell RNA sequencing

Intestinal ILCs were sorted from Chow and TPN mice on day 7 post‐model establishment using the SORP FACSARIA II flow cytometer. The Single Cell 3' Library and Gel Bead Kit (V3.1, 1000121, 10 × Genomics) and the Chromium Single Cell G Chip Kit (1000120, 10 × Genomics) were used to capture cells and synthesize cDNA. We loaded the suspension of cells onto the Chromium Single Cell Controller (10 × Genomics), and single‐cell gel beads were generated in emulsion, following the manufacturer's instructions. Each channel introduces about 10,000 cells. The library preparation of single‐cell RNA sequencing (scRNA‐Seq) was executed following the established protocol by the manufacturer. A minimum depth of 100,000 reads/cell was achieved using the NovaSeq. 6000 platform (Illumina), with paired‐end read lengths of 150 bases. The sequencing services were provided by CapitalBio Technology.

### Statistical analysis of scRNA‐Seq

The analysis of scRNA‐seq data was conducted by CapitalBio Technology, utilizing the CapitalBio Cloud Analysis Platform. The software Cell Ranger (V.7.1.1) was used to facilitate the alignment, count barcodes, filter, and count unique molecular identifiers (UMI). Additionally, clustering was performed using the R package Seurat 3.0. Cells with genes fewer than 200, gene count ranking in the top 1%, or those exhibiting a ratio of mitochondrial genes exceeding 25% were classified as outliers and subsequently excluded from the analysis. Visualization was conducted using UMAP and t‐distributed stochastic neighbor embedding. These analyses focused on the top 20 gene markers of each cluster. R programming package was used to visualize the resulting data. Monocle R package was used to construct single‐cell trajectory plots. Annotation of cell type was performed utilizing SingleR (https://bioconductor.org/packages/devel/bioc/html/SingleR.html), a tool that enables unbiased identification of cell types from scRNA‐seq data.

### Statistics and reproducibility

Clinical data were statistically analyzed by SPSS (version 25, IBM Corp.). Experimental data were analyzed using Prism 10 (GraphPad). For between‐group comparisons of unmatched data, non‐parametric data were analyzed using the Mann–Whitney *U* test, and normally distributed data were analyzed using the Student's *t*‐test. The relationship between *L. murinus* abundance and IL‐22 levels was evaluated using Spearman correlation analysis. For between‐group comparisons involving multiple groups, the Kruskal–Wallis *H* test was used for unmatched data, and normally distributed data were analyzed using the one‐way analysis of variance (ANOVA), followed by post‐hoc correction using Dunnett's *t*‐test. The significance threshold was at less than 0.05 for two‐tailed *p*‐values.

## AUTHOR CONTRIBUTIONS

**Longchang Huang**: Conceptualization; investigation; writing—original draft; methodology; validation; visualization; writing—review and editing; software; formal analysis; project administration; data curation. **Peng Wang**: Conceptualization; investigation; methodology; validation; formal analysis; software; data curation; project administration; visualization. **Shuai Liu**: Investigation; writing—original draft; methodology; validation; visualization; software; formal analysis; data curation. **Guifang Deng**: Investigation; writing—review and editing; data curation; supervision; conceptualization. **Xin Qi**: Validation; methodology; visualization; software; formal analysis; writing—original draft; writing—review and editing. **Guangming Sun**: Data curation; investigation; validation; visualization. **Xuejin Gao**: Data curation; software; formal analysis. **Li Zhang**: Data curation; supervision; formal analysis; project administration; methodology. **Yupeng Zhang**: Data curation; formal analysis; software. **Yaqin Xiao**: Data curation; investigation. **Tingting Gao**: Data curation; project administration. **Gulisudumu Maitiabula**: Data curation; supervision; project administration. **Xinying Wang**: Conceptualization; funding acquisition; writing—review and editing; resources; supervision.

## CONFLICT OF INTEREST STATEMENT

The authors declare no conflicts of interest.

## ETHICS STATEMENT

The Ethics Committee of Jinling Hospital (Nanjing University of Medicine, Nanjing, China) approved the protocol for the clinical study (No. 2021NZKY‐024‐01). All animal procedures complied with the guidelines of the Animal Care and Use Committee of the Jinling Hospital. The Ethics Committee of Jinling Hospital (Nanjing University of Medicine, Nanjing, China) approved the protocol for the animal study (No. 2023JLHGZRDWLS‐00078).

## Supporting information


**Figure S1.** Flowchart summarizing the process of enrolling study participants.
**Figure S2.** TPN‐modulated microbiota mediates intestinal barrier damage.
**Figure S3.** TPN shows no effect on Th17, Th22, and γδT cells.
**Figure S4.** TPN induces a microenvironment with low ILC3 responses.
**Figure S5.** TPN results in a decrease of *L. murinus*.
**Figure S6.** *L. murinus* ameliorates intestinal barrier damage.
**Figure S7.** ICA is critical for the effects of *L.murinus*.
**Figure S8.** ICA promotes the function of ILC3s by targeting Rorγt.

**Table S1.** Clinical characteristics of 338 patients.
**Table S2.** Primer sequences for real‐time PCR used in the study.
**Table S3.** Key resources used in the study.

## Data Availability

The data that support the findings of this study are openly available in GSA at https://ngdc.cncb.ac.cn/bioproject/browse/PRJCA034984, reference number PRJCA034984. All the sequencing data have been deposited in GSA under BioProject accession number PRJCA034984 (https://ngdc.cncb.ac.cn/bioproject/browse/PRJCA034984) and PRJCA035609 (https://ngdc.cncb.ac.cn/bioproject/browse/PRJCA035609). The Metabolomics data reported in this paper have been deposited in the OMIX, China National Center for Bioinformation/Beijing Institute of Genomics, Chinese Academy of Sciences (accession no. OMIX008699, https://ngdc.cncb.ac.cn/omix/release/OMIX008699 and OMIX008700, https://ngdc.cncb.ac.cn/omix/release/OMIX008700). The data and scripts used are saved in GitHub https://github.com/hlc999/HLC_001. Supplementary materials (figures, tables, graphical abstract, slides, videos, Chinese translated version, and update materials) may be found in the online DOI or iMeta Science http://www.imeta.science/.
