# Supplementary material for: Gut microbiota‐derived tryptophan metabolites improve total parenteral nutrition‐associated infections by regulating Group 3 innate lymphoid cells
Source: Imeta. 2025 Feb 26;4(2):e70007. doi: 10.1002/imt2.70007 (PMC11995168; doi:10.1002/imt2.70007)
Supplement: Supplementary file 1 — Figure S1. Flowchart summarizing the process of enrolling study participants. Figure S2. TPN‐modulated microbiota mediates intestinal barrier damage. Figure S3. TPN shows no effect on Th17, Th22, and γδT cells. Figure S4. TPN induces a microenvironment with low ILC3 responses. Figure S5. TPN results in a decrease of L. murinus. Figure S6. L. murinus ameliorates intestinal barrier damage. Figure S7. ICA is critical for the effects of L.murinus. Figure S8. ICA promotes the function of ILC3s by targeting Rorγt. [file IMT2-4-e70007-s001.docx]

**Supporting information to**

**Gut microbiota-derived tryptophan metabolites improve total parenteral nutrition-associated infections by regulating Group 3 innate lymphoid cells**

**Running title:** Tryptophan metabolites enhance TPN immunity

Longchang Huang^1#^, Peng Wang^2#^, Shuai Liu^1#^, Guifang Deng^3#^, Xin Qi^1^, Guangming Sun^1^, Xuejin Gao^1^, Li Zhang^1^, Yupeng Zhang^1^, Yaqin Xiao^1^, Tingting Gao^1^, Gulisudumu Maitiabula^1^, Xinying Wang^1*^

^1^Department of General Surgery, Jinling Hospital, Medical School of Nanjing University, Nanjing 210002, China

^2^Department of Digestive Disease Research Center, Gastrointestinal Surgery, The First People's Hospital of Foshan, Guangdong 510000, China

^3^Department of Clinical Nutrition, Union Shenzhen Hospital of Huazhong University of Science and Technology, Shenzhen 518052, China

^#^These authors contributed equally: Longchang Huang, Peng Wang, Shuai Liu and Guifang Deng

^*^Correspondence: [wangxinying@nju.edu.cn](mailto:wangxinying@nju.edu.cn) (Xinying Wang)

**Supplementary figures**

**
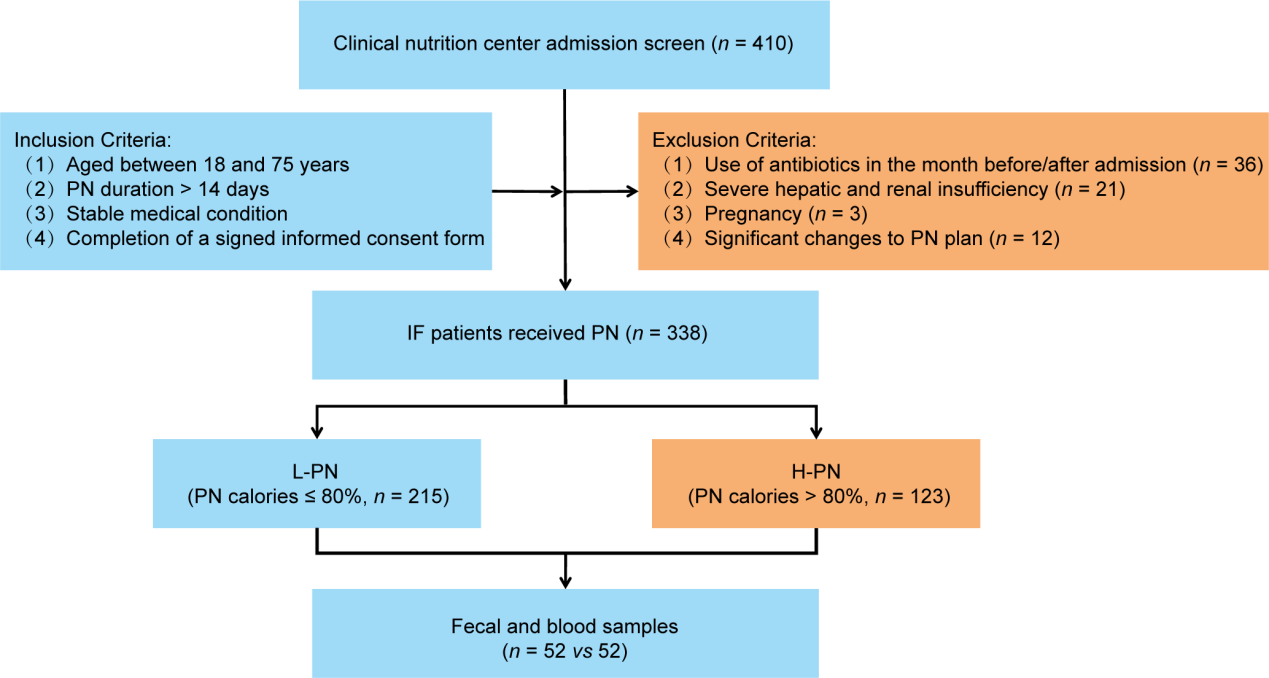
**

**Figure S1 Flowchart summarizing the process of enrolling study participants.** Total 410 patients with CIF were taken in the Clinical Nutrition Center of the Jinling Hospital, Nanjing, Jiangsu, China, from August 2017 to August 2022. 72 patients were excluded according to the exclusion criteria, and 338 patients were included in this study. Finally, 215 patients were assigned to the L-PN group, and 123 were assigned to the H-PN group. One hundred four patients contributed Fecal and blood samples.


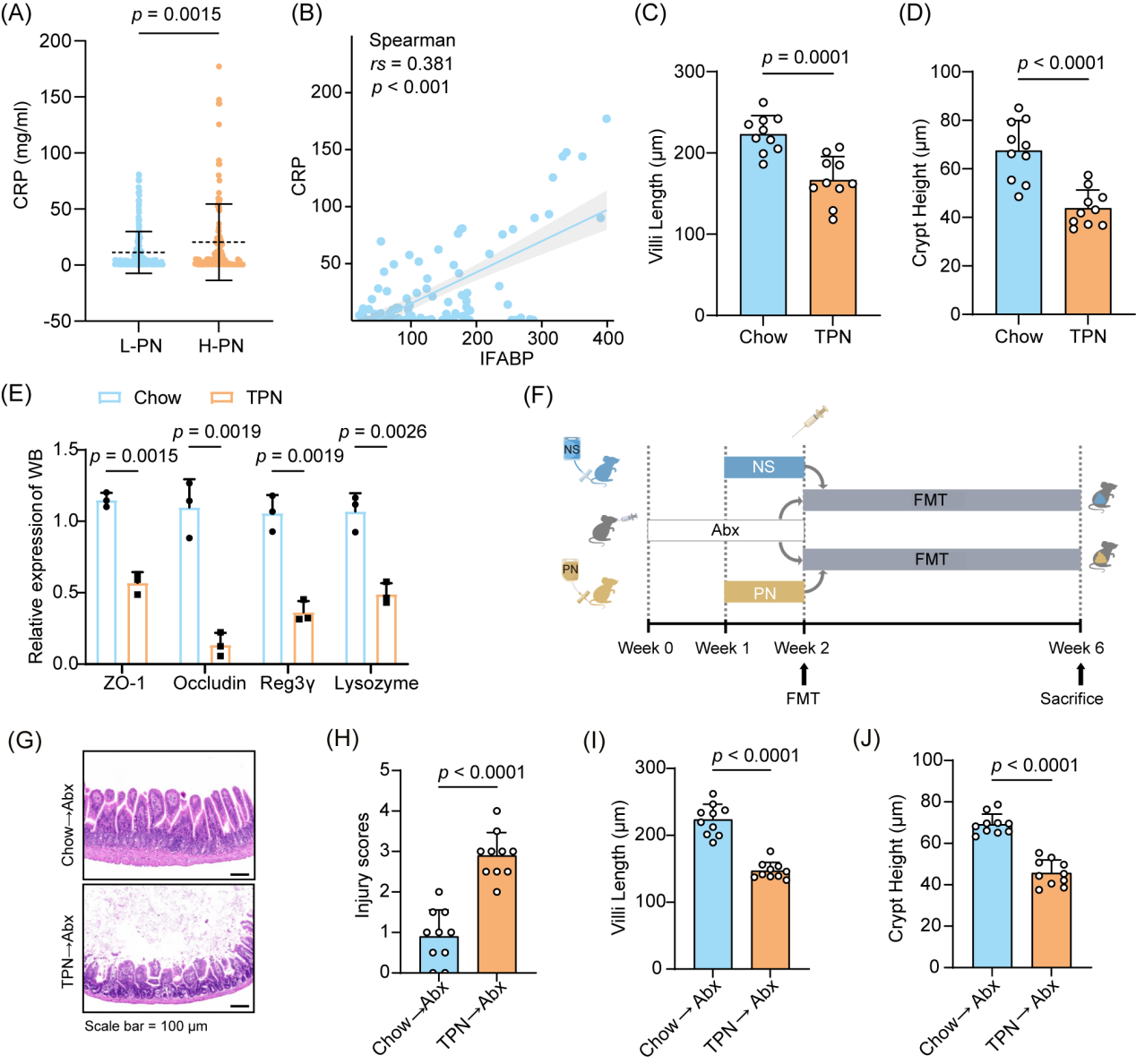


**Figure S2 TPN-modulated microbiota mediates intestinal barrier damage.** (A) Serum levels of CRP in patients with CIF (*n* = 338). (B) Correlation between CRP and IFABP levels in patients (*n* = 104). (C-D) The average villi length and crypt height were compared (*n* = 10 mice per group). (E) The results of Western blot. (F) Experimental schematic of FMT. (G-H) Representative intestinal HE staining (G) and injury scores (H) (*n* = 10 mice per group). (I-J) The average villi length (I) and crypt height (J) were compared in two groups (*n* = 10 mice per group). *p* values were determined by Mann-Whitney U test (A) and the Student’s t-test (C-E and H-J). All statistical tests were two sided.


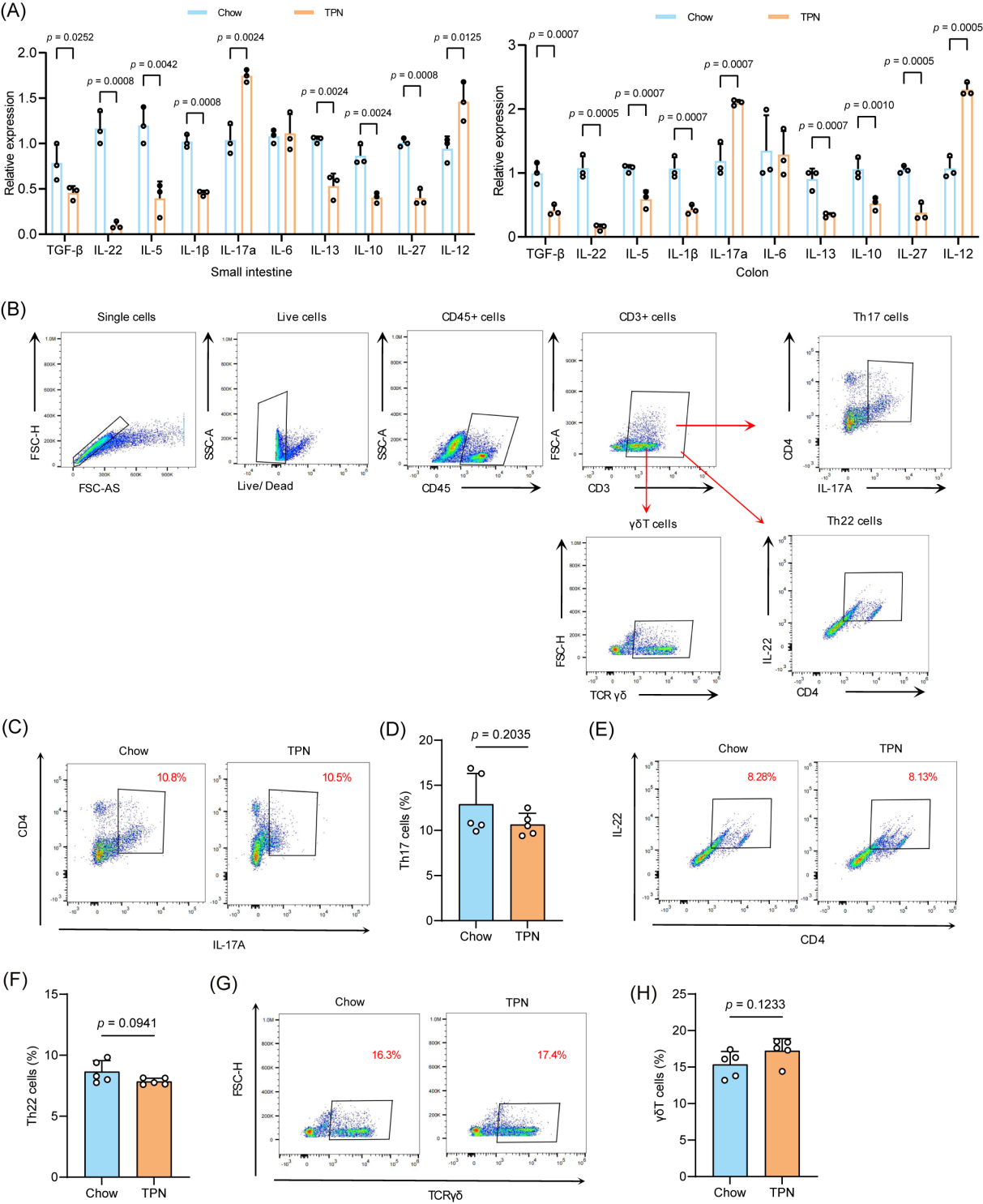


**Figure S3 TPN shows no effect on Th17, Th22 and γδT cells.** (A) q-PCR detects the expression levels of cytokines in the small intestine and colon. (B) Gating strategy for analyses of Th17, Th22, and γδT cells. (C-D) Representative FACS plots (C) and the frequency (D) of Th17 cells. (E-F) Representative FACS plots (E) and the frequency (F) of Th22 cells. (G-H) Representative FACS plots (G) and the frequency (H) of γδ T cells. (*n* = 5 mice per group). *p* values were determined by Mann-Whitney U test (A, D, F, and H). All statistical tests were two sided.


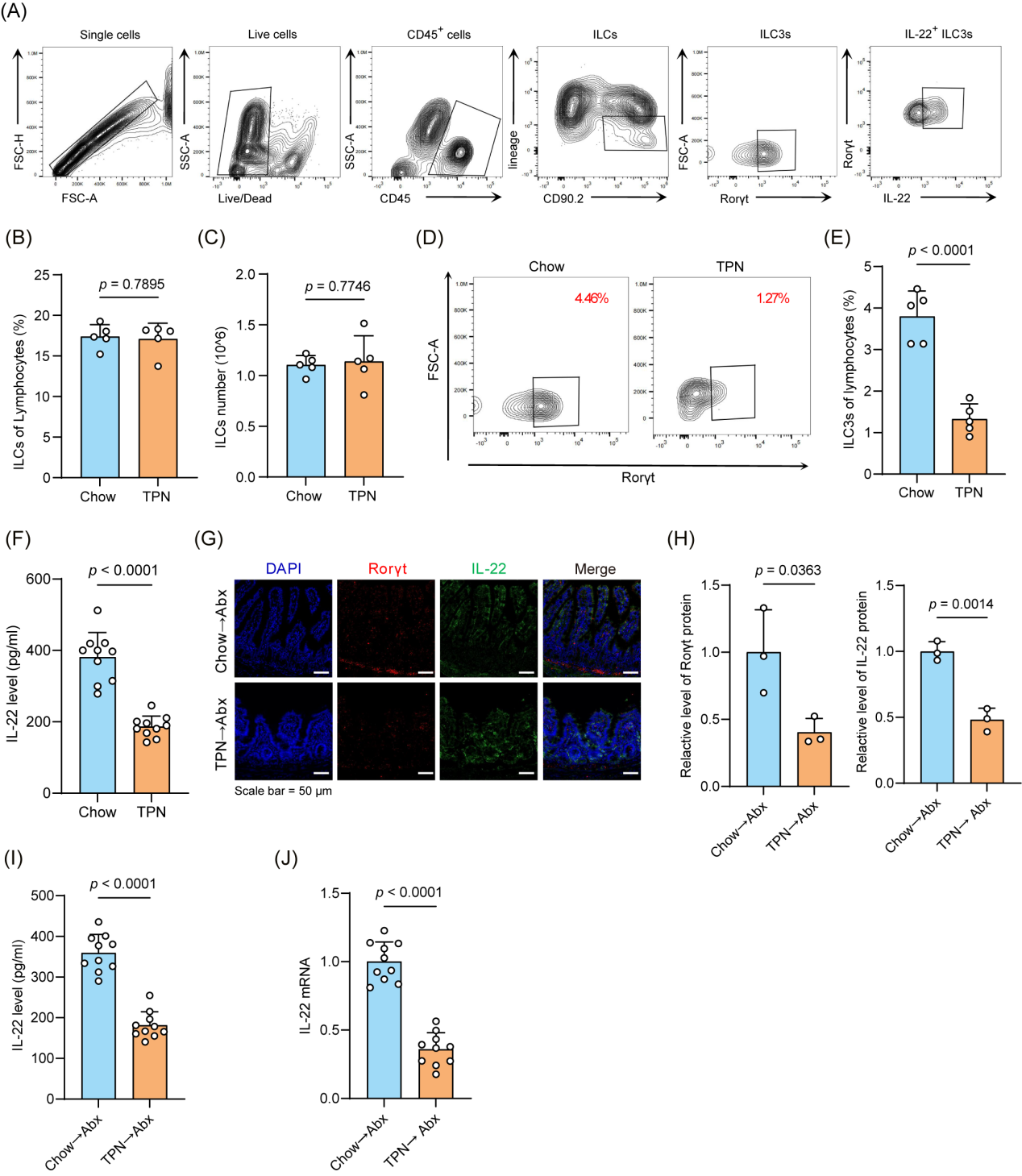


**Figure S4 TPN induces a microenvironment with low ILC3s responses.** (A) Gating strategy for analyses of intestinal ILC3s. (B-C) Percentage (B) and number (C) of intestinal ILCs. (D-E) Representative FACS plots (D) and the frequency (E) of ILC3s. (F) Serum levels of IL-22 in Chow and TPN group mice (*n* = 10 mice per group). (G-H) Immunofluorescence experiments showing Rorγt and IL-22 expression in the intestine of Abx mice after different treatments. (I-J) After different treatments, serum levels (I) and intestinal expression (J) of IL-22 in Abx mice (*n* = 10 mice per group). *p* values were determined by Mann-Whitney U test (B) and the Student’s t-test (C, E-F, and H-J). All statistical tests were two sided.


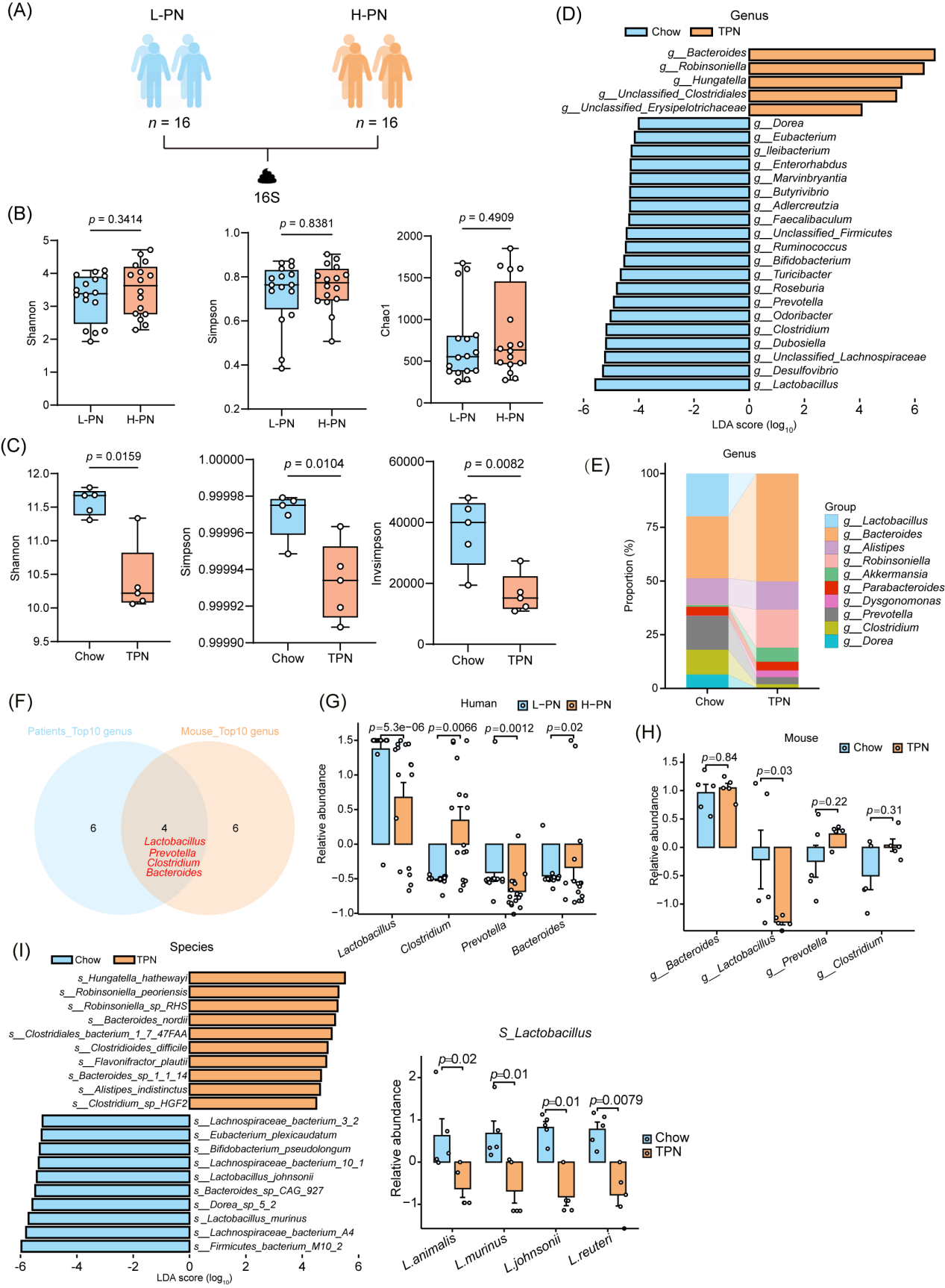


**Figure S5 TPN results in a decrease of *L.murinus*.** (A) Design scheme for the trial. Feces were gathered from the subjects (*n* = 16 individuals/group). (B) Index of Simpson, Shannon, and Chao1 in patients (*n* = 16 individuals/group). (C) Index of Simpson, Shannon, and Invsimpson in mice (*n* = 5 mice per group). (D) LDA at the level of the genus in mice. (E) Top 10 abundant genus in mice. (F) A Veen diagram shows the top 10 abundant genera of humans and mice. (G) Relative expression levels of four bacterial types at the genus level in patients. (H) The relative expression levels of four bacterial types at the genus level in mice. (I) LDA (left) and four types of *Lactobacillus* (right) at the level of species in mice. *p* values were determined by Mann-Whitney U test (B-C and G-I). All statistical tests were two sided.


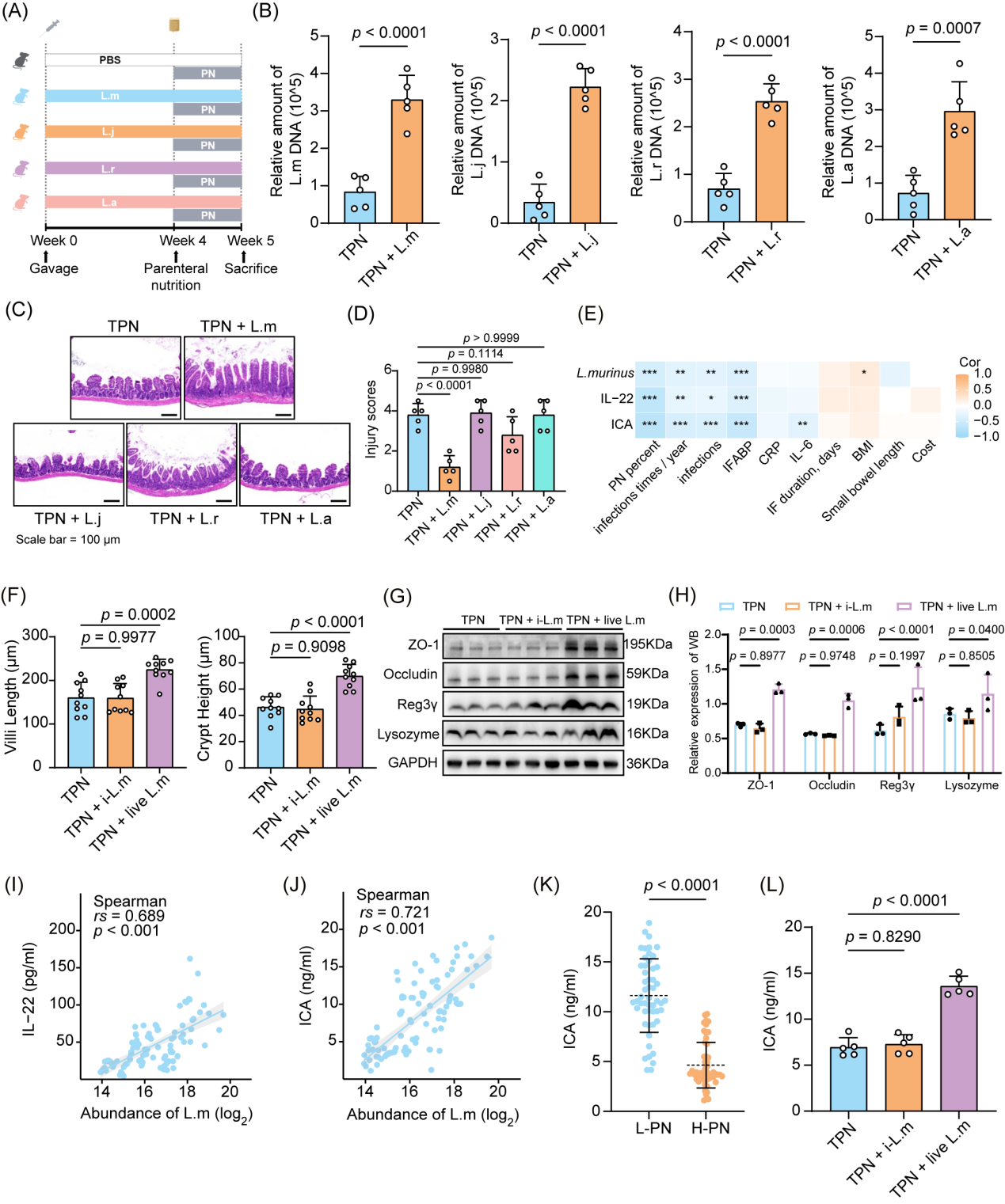


**Figure S6 *L.murinus* ameliorates intestinal barrier damage.** (A) Experimental design scheme for single-strain intervention. (B) The abundance of the four Lactobacillus strains significantly increased after the administration of a single strain as design in Figure S6A. (C-D) Representative intestinal HE staining (C) and injury scores (D) (*n* = 5 mice per group). (E) The Spearman correlation analysis of *L. murinus*, ICA, and IL-22 with the clinical features of patients. (F) The average villi length and crypt height were compared (*n* = 10 mice per group). (G-H) The results of Western blot. (I) The correlation between the serum IL-22 levels and the abundance of *L.murinus* of patients. (J) Spearman correlation analysis between *L. murinus* and ICA. (K-L) Fecal ICA in patients (K) and mice (L). *p* values were determined by the Student’s t-test (B), One-way ANOVA (post-hoc analysis used Dunnett’s t-test; D and L), Kruskal-Wallis H test with correction by Dunnett’s t-test (F and H), and Mann-Whitney U test (K). All statistical tests were two sided.


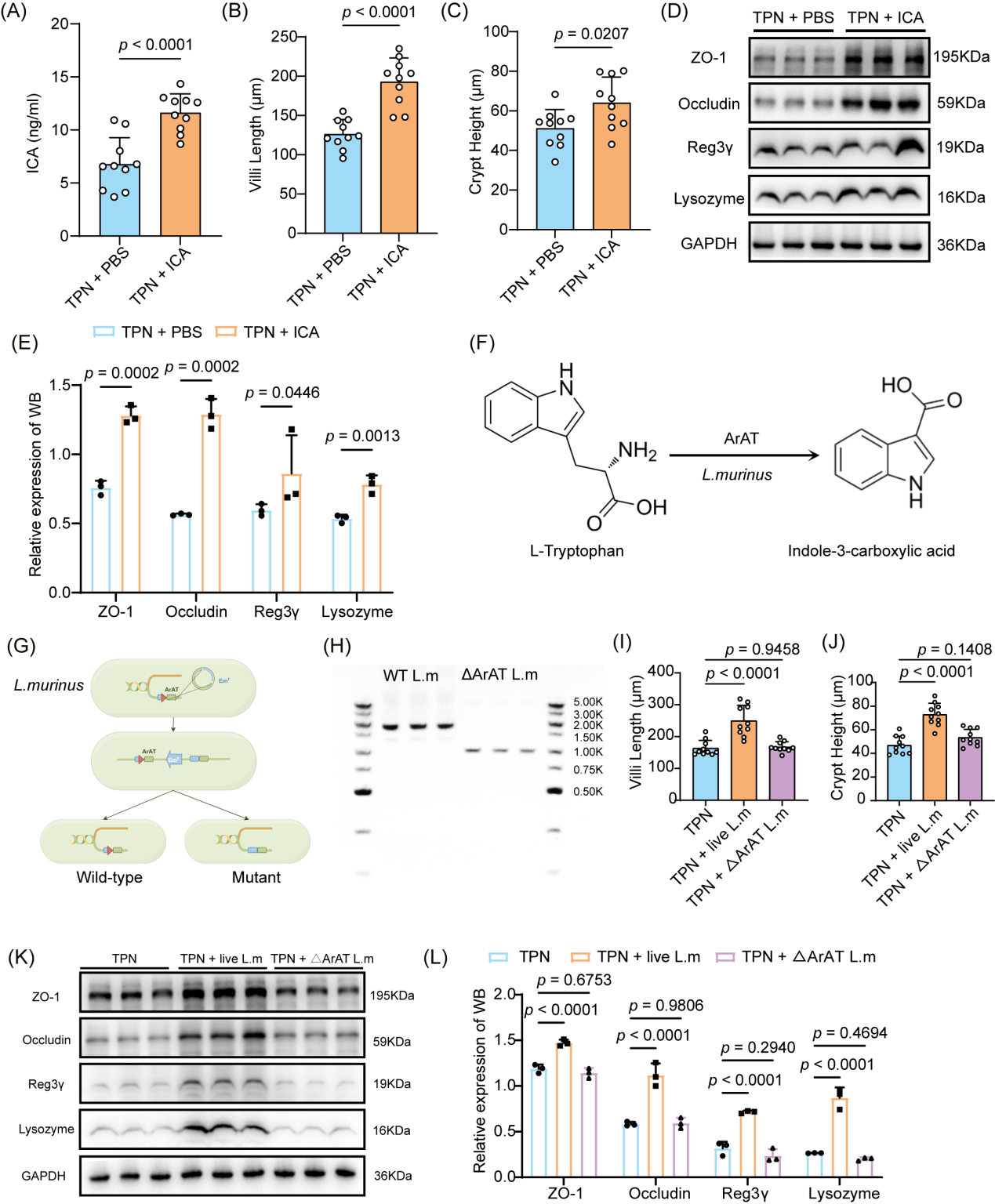


**Figure S7 ICA is critical for the effects of *L.murinus*.** (A) The fecal level of ICA from TPN mice treated PBS control or ICA (*n* = 10 mice per group). (B-C) The average villi length (B) and crypt height (C) were compared (*n* = 10 mice per group). (D-E) The results of Western blot. (F) This metabolic pathway illustrates the production of ICA by the bacterium *L. murinus* from the tryptophan. (G) A schematic depicting the generation of ∆ArAT *L. murinus* by homologous recombination. (H) Verifying the mutant by PCR. (I-J) The average villi length (I) and crypt height (J) were compared (*n* = 10 mice per group). (K-L) The results of Western Blot. *p* values were determined by the Student’s t-test (A-C and E), Kruskal-Wallis H test with correction by Dunnett’s t-test (I), and One-way ANOVA (post-hoc analysis used Dunnett’s t-test; J and L). All statistical tests were two sided.

_
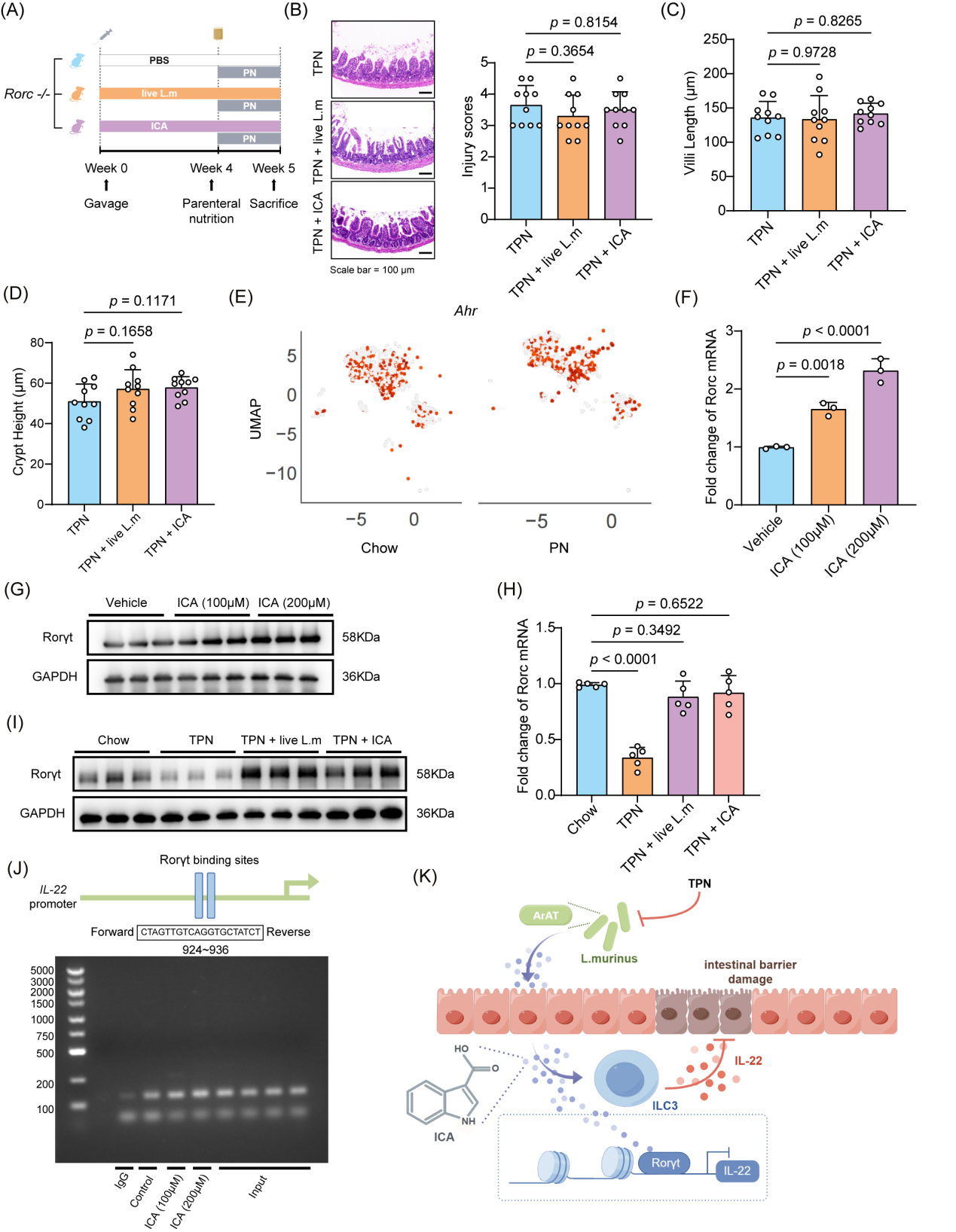
_

**Figure S8 ICA promotes the function of ILC3 by targeting Rorγt.** (A) The design scheme for the experiment of *Rorc*^-/-^ mice (*n* = 10 mice per group). (B) Representative intestinal HE staining and injury scores in *Rorc*^-/-^ mice. (*n* = 10 mice per group). (C-D) The average villi length (C) and crypt height (D) were compared. (*n* = 10 mice per group). (E) The UMAP analysis of NCR^+^ ILC3s reveals no significant differences in the gene expression levels of *Ahr* between the Chow and TPN groups. (F) RT-PCR was conducted to detect the *Rorc* mRNA expression in ILC3s (*n* = 3 replicates per group). (G) ICA promotes the expression of Rorγt in ILC3s. (H) RT-PCR was performed to quantify the *Rorc* mRNA expression in the intestine tissues of mice (*n* = 5 mice per group). (I) ICA promotes the expression of Rorγt in mouse intestinal tissues. (J) ChIP-qPCR was applied to ascertain the effect of ICA on Rorγt's binding to *IL-22*'s promoter. (K) Schematic of the research. The use of TPN causes dysbiosis of the gut microbiota, inhibiting the secretion of IL-22 by intestinal ILC3s, which in turn results in damage to the intestinal barrier. The reduction of *L.murinus* is an important factor in this process. Supplementing with *L.murinus* can increase the expression of ICA. ICA promotes the secretion of IL-22 by ILC3s by targeting Rorγt, thereby improving the intestinal barrier and reducing susceptibility to infections. *p* values were determined by Kruskal-Wallis H test with correction by Dunnett’s t-test (B) and One-way ANOVA (post-hoc analysis used Dunnett’s t-test; C-D, F, and H). All statistical tests were two sided.
